# Diagnosis and Treatment of Autoimmune Hepatitis: Questions, Answers, and Illustrative Cases: Endorsed by Autoimmune Liver Diseases Special Interest Group, Turkish Association for the Study of Liver

**DOI:** 10.5152/tjg.2023.23242

**Published:** 2023-11-08

**Authors:** Ersan Özaslan, Fulya Günşar, Aslı Çiftçibaşı Örmeci, İbrahim Hatemi, Cumali Efe, Gülen Akyol, Nesrin Turhan, Funda Yılmaz Barbet, Özgül Sağol, Çiğdem Ataizi Çelikel, Mine Güllüoğlu, Dilara Turan Gökçe, Zülal İstemihan, Tuğçe Eşkazan, Ramazan İdilman

**Affiliations:** 1 Department of Gastroenterology, University of Health Sciences, Ankara Bilkent City Hospital, Ankara, Turkey; 2 Department of Gastroenterology, Ege University, İzmir, Turkey; 3 Department of Gastroenterology, İstanbul University Çapa Faculty of Medicine, İstanbul, Turkey; 4 Department of Gastroenterology, İstanbul University Cerrahpaşa Faculty of Medicine, İstanbul, Turkey; 5 Department of Gastroenterology, Harran University, Şanlıurfa, Turkey; 6 Department of Pathology, Gazi University, Ankara, Turkey; 7 Department of Pathology, University of Health Sciences, Ankara Bilkent City Hospital, Ankara, Turkey; 8 Department of Pathology, Ege University, İzmir, Turkey; 9 Department of Pathology, Dokuz Eylül University, İzmir, Turkey; 10 Department of Pathology, Marmara University, İstanbul, Turkey; 11 Department of Pathology, İstanbul University Çapa Faculty of Medicine, İstanbul, Turkey; 12 Department of Gastroenterology, Ankara Bilkent City Hospital, Ankara, Turkey; 13 Department of Gastroenterology, Ankara University, Ankara, Turkey

**Keywords:** Autoimmune hepatitis, primary biliary cholangitis, primary sclerosing cholangitis, overlap, variant, treatment

## Abstract

Autoimmune hepatitis (AIH) is a rare, immune-mediated liver disease. It has a heterogeneous nature with varied clinical presentations. The management of patients with AIH is challenging in many ways. The main difficulties are inexperience due to the rarity of the disease, diagnostic confusion in controversial areas such as variant/overlap cases, acute presentations, the presence of non-alcoholic fatty liver disease or drug-induced liver injury features, and the long and complex course of treatment. Here, we provide a clear, concise, and visualized review regarding the diagnosis and treatment of AIH, including illustrative cases.

**Main Points**

- Autoimmune hepatitis (AIH) has various clinical presentations and many features overlap with other autoimmune (primary biliary cholangitis and primary sclerosing cholangitis) or common liver diseases (non-alcoholic fatty liver disease and drug-induced liver injury).
- Since there is no pathognomonic criterion of AIH, it is diagnosed by the combination of characteristic clinical and pathological features.
- Although the treatment process is difficult, remission is usually achieved, but there is no curative treatment.
- In this review, we present the issues in clinical practice related to the diagnosis and treatment of AIH as well as many illustrative case examples.

## Introduction

Autoimmune hepatitis (AIH) is a rare, immune-mediated inflammatory liver disease that is characterized by circulating autoantibodies, hypergammaglobulinemia and typical liver biopsy findings of interface hepatitis with lymphoplasmacytic infiltration.^1^ In classical teaching, an AIH patient is defined as a middle-aged female with chronic hepatitis, presenting with nonspecific symptoms such as fatigue, but actually, it is a very heterogeneous disease with varied clinical presentations and affecting people of all ages and both genders. Although it typically responds to immunosuppression, the treatment process is long, requires fine adjustment, is associated with serious side effects, and is not curative.

Many points in AIH management are not properly applied in clinical practice as reflected in real-world data.^2,3^ The main reasons for this are inexperience due to the rarity of the disease, diagnostic confusion in controversial areas such as variant/overlap cases, acute presentations, the presence of non-alcoholic fatty liver disease (NAFLD) or drug-induced liver injury (DILI) features, and difficulties in the long-lasting and challenging treatment process. Moreover, evidence-based knowledge is scarce in many areas regarding AIH. In this review, we tried to answer frequently asked questions in daily clinical practice regarding AIH, focusing only on the diagnosis and treatment. Since our aim was a clear, concise, and visualized AIH review, therefore, the current international guidelines, position papers, and reviews were selected as main references. After giving a short summary in the main headings, we described the clinical problems in a question–answer format. If needed, for complex concepts, an explanation section was also added. To make the topics more understandable, short-case vignettes were presented. 

## Clinical Presentation

**Figure 1.**
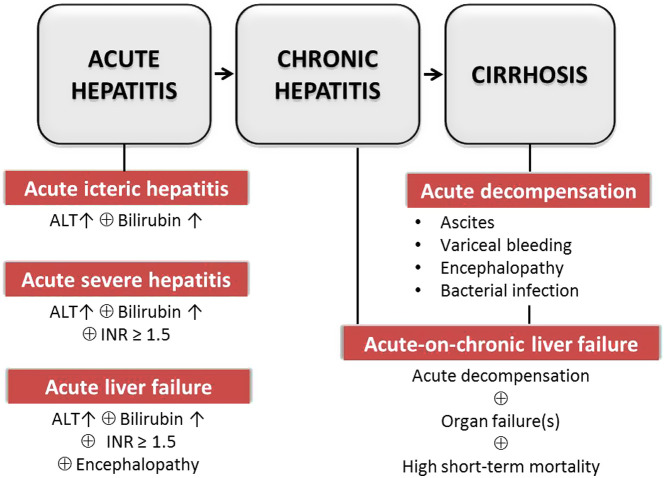
The clinical spectrum of autoimmune hepatitis.

Autoimmune hepatitis is more common in women than men (3 : 1), with a bimodal distribution of presentation peaks in the teenage years and in middle age between the fourth and sixth decade.^1,4^ Autoimmune hepatitis can be diagnosed also in elderly patients older than 70 years old. The type of presentation is similar to younger patients, although half of them are cirrhotic. Response to treatment is similar with fewer relapses and without differences regarding adverse events.^5^ Autoimmune hepatitis can present as many forms of acute or chronic liver disease, symptomatic or asymptomatic (Figure 1). Patients with chronic hepatitis have non‐specific symptoms that may include fatigue, malaise, anorexia, right upper quadrant pain, weight loss, amenorrhea, and polyarthralgia, while in the case of cirrhosis, the signs of palmar erythema, spider angioma, splenomegaly, ascites, pedal edema, or encephalopathy may be seen. Acute cases usually present as acute viral hepatitis-like illness, but the features of hepatic and other organ failure(s) (OF) are also added in severe presentations, such as acute liver failure (ALF) and acute-on-chronic liver failure (ACLF).^6^

### What Are the Main Clinical Presentations of Autoimmune Hepatitis?

The clinical presentations of AIH can be divided into 3 main groups, acute hepatitis, chronic hepatitis, and cirrhosis, at approximately similar rates.^1,4^ The clinical course of AIH is chronic and fluctuating. Cirrhosis at initial presentation in some AIH patients can be explained by subclinical episodes of flare and spontaneous remission.

### What Are the Subtypes of Acute Autoimmune Hepatitis Presentations?

The term “acute” refers to both disease duration (<6 months) and a sudden marked increase in transaminase levels (>10 fold). So, in addition to “truly acute AIH regarding timing,” if the transaminases are significantly higher (>10 fold) in chronic AIH with subclinical course, it is also initially evaluated as acute AIH instead of “acute-on-chronic AIH.” Conversely, some cases of chronic AIH may have signs of sudden clinical deterioration without excessive transaminase elevation (acute-on-chronic AIH subtypes are described later). Actually, at first presentation, the most definitive method to differentiate between acute and acute-on-chronic AIH is liver biopsy, in which the presence of fibrosis favors chronicity. However, it is not usually required for the diagnosis of “acute” scenarios in patients with a previous diagnosis of AIH.

Acute AIH subgroups are defined as acute icteric hepatitis, acute severe hepatitis (ASH), and ALF, while acute-on-chronic AIH groups include acute decompensation (AD) and ACLF (Figure 1).

### How Do You Define Acute Liver Failure and What Are the Peculiar Features of Autoimmune Hepatitis-Induced Acute Liver Failure?

**Acute liver failure is characterized by severe liver damage with **international normalized ratio (INR) ≥1.5 and encephalopathy in a patient without existing liver disease over a period of <26 weeks. **Autoimmune hepatitis-induced ALF may be acute but it is usually subacute in presentation and compared to chronic AIH, the probable absence of autoantibodies and/or lack of serum immunoglobulin G (IgG) increase, difficulties in obtaining a liver biopsy due to coagulopathy, and probable triggers such as drugs and viruses may lead to underdiagnosis or a late diagnosis. Clinical clues, extending and repeating serological tests, alternative routes for liver biopsy such as transjugular approach, flexible use of AIH scoring systems, and some radiological clues such as heterogeneous hypoattenuation are required to arrive at a diagnosis. Early diagnosis and timely immunosuppression may save many patients. On the other hand, the imaging appearance of subacute AIH-ALF may simulate cirrhosis and erroneously assign these patients to a lower priority on the waiting list and may also lead to the withholding of life-saving steroid-based therapy. Detailed medical history and the demonstration of severe necroinflammation without fibrosis in liver biopsy favor the diagnosis of ALF.**

Considering jaundice as the first symptom of developing encephalopathy, ALF categorization may be simplified as hyperacute (<7 days), acute (<1 month), and subacute (<6 months). Acetaminophen toxicity, idiosyncratic drug reactions, and hepatitis viruses are the most common causes of ALF.^7^ Autoimmune hepatitis is an increasingly recognized cause of ALF either on its own or as a probable cause in over half of the cases in the indeterminate ALF group.^8,9^ It may be acute but usually subacute in presentation, and compared to chronic AIH, failure to reach detectable serum antibody and/or IgG levels and difficulties in obtaining a liver biopsy may lead to underdiagnosis.^6,9^ As discussed in the diagnosis section, the history of autoimmune disorder, extending and repeating serology for the presence of antibodies and/or increased IgG, alternative routes for liver biopsy, and flexible use of AIH scoring systems can provide enough data. In a severe case, due to the importance of early therapy before the development of encephalopathy, a diagnostic steroid trial is rather justified even before obtaining the results of autoimmune serology and liver biopsy.^10,11^

Radiology may also have some pearls and pitfalls in such cases. The histological characteristic of acute onset AIH is its ‘‘heterogeneity’’ especially in ALF, which corresponds with its radiological and clinical heterogeneity.^8^ The pearl is that unenhanced computerized tomography (CT) often shows heterogeneous hypoattenuations reflecting histological massive hepatic necrosis.^12^ On the other hand, the pitfall is that the imaging appearance of subacute AIH-ALF may exhibit various abnormalities simulating cirrhosis from surface nodularity to evidence of portal hypertension like ascites and splenomegaly. Access to medical history and the demonstration of bridging necrosis instead of bridging fibrosis with or without newly forming regenerative nodules in liver biopsy may clear the dilemma in favor of ALF over cirrhosis.^6^

### How Can We Differentiate Acute Decompensated Cirrhosis and Acute-on-Chronic Liver Failure in Patients with Autoimmune Hepatitis?

**Unlike ALF, acute decompensated cirrhosis and ACLF develop in the setting of chronic liver disease. Acute decompensation defines the acute development of traditional complications such as ascites, encephalopathy, gastrointestinal bleeding, or bacterial infection in cirrhosis. Acute-on-chronic liver failure refers to a severe form of AD in chronic liver disease, associated with single or multiple-OF(s), and high risk of short-term mortality. Acute-on-chronic liver failure is driven by intense systemic inflammation which is triggered by the precipitating factor(s) which may be either hepatic (i.e., heavy alcohol intake, viral hepatitis, DILI, and autoimmune hepatitis) and/or extrahepatic (i.e., infections and surgery). Acute-on-chronic liver failure diagnosis is made according to the scoring systems such as The Asian Pacific Association for the Study of Liver, Research Consortium Score (APASL-AARC), European Association for the Study of the Liver, Chronic Liver Failure Consortium (EASL-CLIF-C), and The North American Consortium for the Study of End Stage Liver (NACSELD) established by international societies.**

Acute-on-chronic liver failure is a distinct entity different from classical AD of cirrhosis.^13,14^ Traditionally, the natural history of cirrhosis is characterized by an asymptomatic compensated phase followed by a decompensated phase. Depending on the intensity of inflammation, the decompensation type covers a spectrum ranging from simple decompensation to ACLF. Acute decompensation defines the acute development of ascites, encephalopathy, gastrointestinal bleeding, bacterial infection, or any combination of these complications. Bacterial infection may precipitate and/or constitute part of the AD process. Compensated cirrhosis defines the disease phase prior to the first AD. Decompensated cirrhosis defines the disease phase after the first AD. Acute-on-chronic liver failure is the most severe entity across the spectrum, including OF(s) and the high risk of short-term mortality. Its definition varies among the international societies, while the early, late, and advanced stages of the ACLF syndrome may be assessed by APASL, EASL, and NACSELD criteria, respectively.^15-17^ While the definition of APASL includes acute severe organ dysfunctions as ACLF in both chronic hepatitis and cirrhotic patients, EASL and NACSELD define ACLF only in cirrhosis.

In clinical practice, ACLF is recognized by the presence of chronic liver disease along with an elevation in the serum bilirubin and prolongation of the INR, regardless of the magnitude of the increase in serum transaminase levels. The presence of OF(s) supports the diagnosis.^18^ The failure of one or more of the 6 major organ systems (liver, kidneys, brain, coagulation, circulation, and respiration) is evaluated by EASL-CLIF-C scoring system. In patients with acutely decompensated cirrhosis and no ACLF, CLIF-C AD score, Model for End-Stage Liver Disease (MELD) score, and MELD-Na score have similar abilities to predict the occurrence of ACLF and all perform better than the Child–Pugh score.^14^ Asian Pacific Association for the Study of Liver and NACSELD have their own scoring systems for ACLF. All of the mentioned scoring systems are available online.^18^

In recent EASL-ACLF guidelines,^14^ AIH is mentioned among the rare precipitants of ACLF. Non-adherence or de-escalation to immunosuppressive therapy, postpartum period, and severe exacerbation of undiagnosed or misdiagnosed AIH are listed as probable causes of AIH-induced ACLF. Of course, common precipitants such as infection, DILI, or severe GI hemorrhage can also trigger ACLF in an already diagnosed AIH patient. Therefore, clinical and laboratory findings should be evaluated to reveal the precipitating factor, while diagnosing ACLF with scoring systems.

The basic concepts of ACLF have been largely derived from clinical studies conducted in patient groups such as alcoholic liver disease, Hepatitis C virus (HCV), and Hepatitis B virus (HBV), but the general results can also be applied to the AIH group. Since AIH-related ACLF has been evaluated together with ALF cases under titles such as acute, acute severe, and acute-on-chronic in previous studies, it is not possible to reach specific diagnostic and therapeutic approaches, further studies involving homogenized ACLF subgroups are needed. The existing literature is summarized in the section “Treatment.”

## Diagnosis

**Figure 2.**
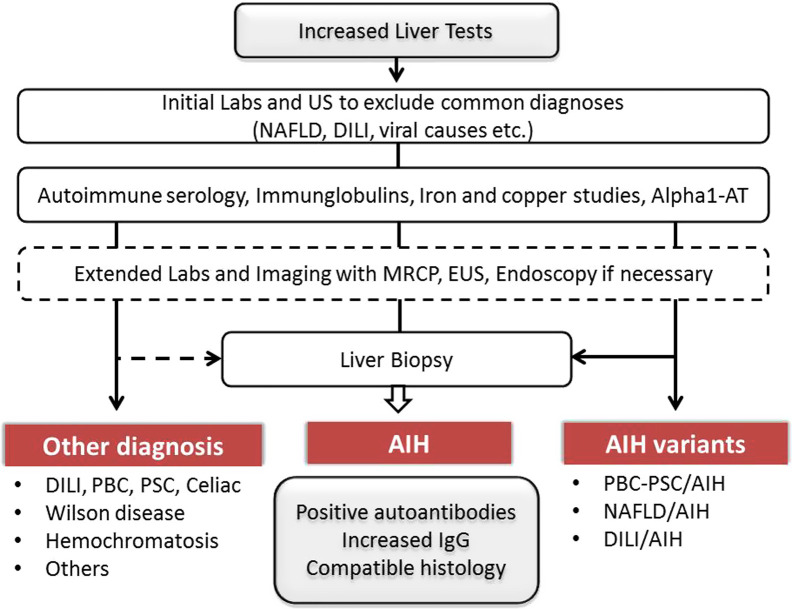
The diagnostic approach to autoimmune hepatitis .

The diagnosis of AIH is usually suspected because of clinical symptoms, abnormal liver tests, or positive autoantibodies. The characteristic features are elevated serum transaminases, elevated serum IgG level, positive serological marker(s), and suggestive histology.^1,4^ Autoimmune hepatitis lacks a signature diagnostic marker, so the diagnosis requires the combination of characteristic features and the exclusion of other diseases (Figure 2). Extended serological/histological evaluation, expert consult and longitudinal follow-up may be needed in challenging cases of AIH variants and seronegative AIH, as described in the following sections.

## Liver Tests

### How Do We Interpret the Magnitude of Serum Transaminase Elevation in Autoimmune Hepatitis?

The characteristic biochemical feature of AIH is serum transaminase elevation including ALT and AST, which are imperfect markers of hepatocellular damage.^19^ There may be varying degrees of serum transaminase elevation in AIH. In acute cases, while transaminase levels are usually more than 300 IU/mL, they can increase up to 50 times higher. Conversely, transaminases may be normal in subclinical or cirrhotic AIH cases.^19-21^

In the classic chronic AIH patient, ALT is greater than aspartate aminotransferase (AST), but AST is higher in cirrhotic patients. In very acute cases where transaminases can increase up to 1000s, AST may be higher than ALT in the first days depending on factors such as the half-life and clearance of serum enzymes, and later ALT becomes predominant during the stabilization period.

### What Is the Value of Other Initial Lab Tests for the Diagnostic Work-up?

Serum alkaline phosphatase (ALP) and gamma-glutamyl transferase (GGT) levels are usually normal or slightly increased. At presentation, only 20% of patients with classical AIH have serum ALP levels >2-fold but no more than 4-fold, while serum GGT levels never exceed 5-fold.^22^ Elevated ALP or GGT levels may be due to incomplete/delayed response or an alternative diagnosis such as overlap syndrome.^23^

The serum bilirubin level is normal in the classic AIH patient but increased in acute icteric hepatitis and in severe scenarios such as ALF, ACLF, and decompensated cirrhosis. Severe cases with ALF or ACLF may display other abnormalities such as lactic acidosis, hypoglycemia, hyperammonemia, elevated creatinine, electrolyte abnormalities, hypoxia, and abnormalities in inflammatory markers such as elevated C-reactive protein and neutrophilia.^7,15^

## Serum Immunoglobulins

Approximately 90% of AIH patients have an elevation of serum IgG levels, mostly as a selective elevation of IgG with normal or mildly elevated IgA and IgM. A predominant increase in IgM for PBC and IgA for NAFLD is typical, while IgG, IgA, and IgM are all elevated in cirrhosis of any cause.^1,24^

### How Could We Approach Autoimmune Hepatitis Diagnosis in a Case with High Clinical Suspicion but Normal Serum Immunoglobulin Level?

About 10%-15% of chronic and up to 39% of acute AIH cases may have normal IgG at baseline.^25^ The “normal” range of IgG is wide, so it is not practical to establish the “real normal ranges” of the respective population. When IgG is normal, failure to consider AIH in the differential diagnosis may lead to diagnostic delays and catastrophic results, especially in acute severe scenarios. Therefore, in such cases with “normal” IgG levels, testing for autoantibodies and, if positive, liver biopsy should be performed to confirm or exclude AIH.^26^

## Autoantibodies

Autoimmune serology can be positive in up to ≥95% of AIH cases if testing has been performed according to the International Autoimmune Hepatitis Group (IAIHG) guidelines, but, this is not the case under real-life circumstances in routine clinical laboratories.^26^ According to the autoantibodies detected, AIH is classified into AIH-type 1 or AIH-type 2. Patients with AIH-1 have detectable anti-nuclear autoantibodies (ANAs) and/or smooth muscle autoantibodies (SMAs). Patients with AIH-2 have detectable anti-liver kidney microsomal type-1 (anti-LKM1) or rarely anti-liver kidney microsomal type-3 (anti-LKM3) and/or anti-liver cytosol type-1 (anti-LC1) antibodies. Antibodies against soluble liver antigens/liver pancreas autoantigen (anti-SLA/LP) can also be detected, mainly in AIH-1 patients. While AIH-1 can be seen at any age, AIH-2 is generally the disease of the pediatric group, which has a more severe course and is more difficult to treat.^1,4,26-29^

Indirect immunofluorescence (IIF) on fresh-frozen substrates from combined rodent liver, stomach, and kidney sections is an ideal method for ANA, SMA, anti-LKM-1 and anti-LC1 antibodies, and molecular-based assays (ELISAs and/or western blot) should be used for the investigation of anti-SLA/LP antibodies. However, routine laboratories do not perform IIF testing on triple rodent substrates but rely on IIF on HEp-2 cells and/or commercial ELISAs for ANA, SMA, and LKM-1. The laboratory should adhere to the guidelines both regarding the assays used and suggested cutoff for positivity and this information should be provided clearly to the clinicians.^26,30^

If clinical suspicion persists in the case of a negative initial panel, repeating investigation in reference laboratory for conventional and non-standard autoantibodies (Perinuclear Anti-Neutrophil Cytoplasmic Antibodies/Anti-Neuronal Nuclear Antibodies [pANCA/ANNA], anti-dsDNA and anti-SLA/LP, anti-LKM-1, anti-LKM-3, anti-LC1, anti-F-actin, anti-Ro52, anti-alpha-actinin; AMA and PBC-specific ANA’s sp100 and gp210; and tissue transglutaminase) is recommended.^4,26^ Seropositivity for one of these autoantibodies could support the diagnosis of AIH and lead to liver biopsy or suggest other diagnoses such as PBC, PSC, celiac disease, etc.

### What Is the Main Message Regarding the Sensitivity and Specificity of Autoimmune Hepatitis-Related Autoantibodies?

Among the autoantibodies used in the diagnosis of AIH, ANA has the highest sensitivity but low specificity, while SLA has the highest specificity but low sensitivity. Due to lack of specificity, ANAs are present in a variety of hepatic (NAFLD, DILI, viral hepatitis, etc.) and extra-hepatic (Hashimoto thyroiditis, celiac disease, etc.) diseases and in healthy adults.^31,32^ It should be noted that there is no pathognomonic serologic marker for AIH. Therefore, autoantibody positivity should always be evaluated together with clinical, laboratory, and histological findings. Similarly, autoantibody negativity does not preclude a diagnosis of AIH in the presence of other supporting features.^1,4,27,28^

### What Is the Diagnostic Value of Positive Autoantibody Number and Titer in Autoimmune Hepatitis?

Approximately, half of the AIH patients have multiple markers, usually ANA and anti-smooth muscle autoantibodies (ASMA) together, while the other half have isolated antibodies.^33^ In scoring systems, the probability of AIH diagnosis increases depending on the number and titer of positive markers. Their impact on disease severity and treatment response is less clear.

### Is There a Specific Staining Pattern for Autoantibodies in Autoimmune Hepatitis?

Neither the staining pattern, nor the identification of the target-autoantigens have any specific clinical implication in patients with AIH.^26^ However, PBC-specific ANAs should deserve a special mention in screening that correspond to rim-like nuclear membranes and multiple nuclear dots on IIF using HEp-2 as substrate and gp210 and sp100 antigens in ELISA, with a sensitivity of 30% and specificity of 99%.^34,35^ Their presence warrants investigation for PBC/AIH variant syndromes in AIH patients with cholestatic features.

## Histology

Liver biopsy is the most important component to confirm the diagnosis of AIH. It is also very valuable in determining the grade and stage of the disease, in the differential diagnosis (PBC, PSC, overlap/variant, drug, Wilson, etc.), in highlighting any concomitant disease and differentiation of acute onset vs. flare-up in chronic disease. Liver biopsy enables the evaluation of histological remission during the treatment process.^1,4,27,28,36^

### Is There a Pathognomonic Histologic Feature in the Liver Biopsy for the Diagnosis of Autoimmune Hepatitis?

**No, the interface hepatitis and its components emperiopolesis, plasma cell infiltration, and hepatocellular rosette formation are known characteristic features of AIH, but even these are not pathognomonic. Varying degrees of hepatocyte loss in the form of apoptosis, focal necroinflammation, or confluent necrosis are usually seen. Centrilobular necroinflammation, a form of confluent necrosis with inflammation, may indicate the acute onset or acute flare of chronic AIH, which is also not pathognomonic.**

**Figure 3.**
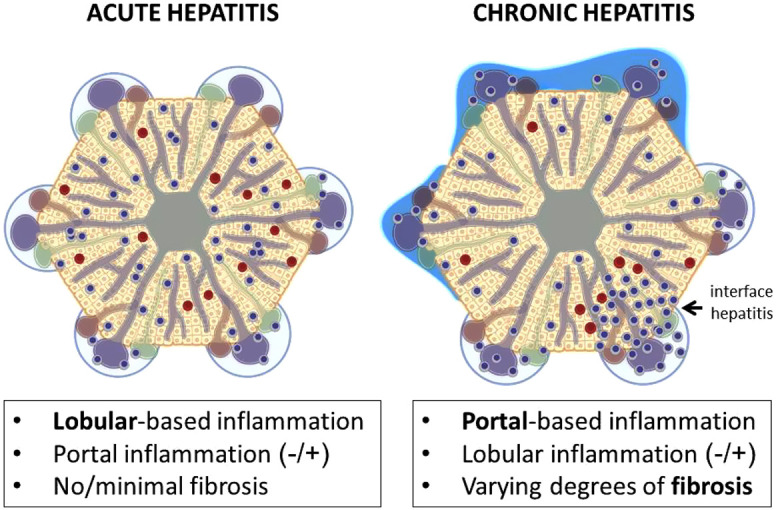
The illustration of characteristic histological findings in acute and chronic hepatitides (red circles, apoptotic hepatocytes; blue zones, fibrosis).

As a histological generalization, acute hepatitis is characterized by lobular predominant inflammation and the absence of fibrosis (lobular inflammation, +/− portal inflammation, and no/minimal fibrosis), while chronic hepatitis is characterized by portal-predominant inflammation and varying degrees of fibrosis (portal-based inflammation, +/− lobular inflammation, and varying fibrosis) (Figure 3). While AIH usually shows signs of chronic hepatitis, it is frequently accompanied by histologic findings of acute hepatitis, especially during clinical exacerbation.^37^ The histological picture may not always correlate with the clinical picture. For example, in a considerable number of cirrhotic patients, histological activity is significant despite normal ALT and IgG.^38^ The severity of the disease is determined mainly by 2 factors: (i) the extent of fibrosis mainly in association with periportal activity due to interface hepatitis and (ii) several forms of confluent necrosis such as centrilobular, bridging, multiacinar, or even submassive/massive necrosis. The latter issue is especially relevant for acute severe presentations such as AIH-related-ASH, ALF, or ACLF.^6^ The extent and severity of necroinflammation and fibrosis are usually scored by using a modified Knodell system which is used for other chronic hepatitis.

There is no single elementary histological criterion that distinguishes classic AIH cases from other causes of chronic/acute hepatitis and early stages of chronic biliary diseases; however, the prominence of plasma cells, the severity of interface hepatitis and the presence of centrilobular confluent necrosis favor AIH diagnosis.^39^ Common A/B/C viral causes of hepatitis can easily be differentiated by serological tests, while acute Hepatitis E virus (HEV) with AIH features may deserve special attention.^40^ Differential diagnosis of other diseases such as early stage PBC or PSC and also drug-induced liver injury may be difficult. This is especially true in scenarios where AIH autoantibodies are negative, IgG is normal, or the presence of isolated centrizonal/lobular activity on histology. In such cases, the presence of plasma cells even if in small numbers, short steroid trial, and advanced histological evaluation for the exclusion of other causes (examination for chronic biliary changes, performing CK7, CK19 stains) may lead to the correct diagnosis.^41,42^ The presence of prominent plasma cells not only in the portal tracts but also within the lobules, especially around areas of focal necrosis or confluent necrosis, is accepted as a valuable clue in favor of AIH. Details of this discussion are provided in the acute AIH and in the section “Overlap/Variant Syndromes.”

Centrilobular necrosis (CN) deserves special attention as it is popular in the hepatology literature, indicating acute injury of groups of hepatocytes followed by their drop out from the hepatocyte cords as a sign of either histological activity or acuity. In fact, as stated before, CN is a type of confluent necrosis and can be seen not only in AIH but also in many different conditions.^4,8,39,42^ It is especially valuable in the diagnosis of seronegative or acute AIH scenarios, but the differential diagnosis must be made from other possible causes of CN such as *idiosyncratic or other forms of DILI* (by history),* viral causes* (by serology), and various forms of vascular injury (by history and clinical clues).

Surprisingly, “lymphocytic cholangitis,” a type of nondestructive inflammatory bile duct injury that is mostly seen in the early stages of PBC, has been repeatedly reported in 24%-83% of AIH patients.^43,44^ The presence of nondestructive biliary inflammation does not exclude the histologic diagnosis of AIH.^45^ The details of this subject are presented in the section “Overlap/Variant Syndromes.”

### Can Cholestasis Be Seen in Autoimmune Hepatitis?

**Autoimmune hepatitis is typically characterized by chronic and/or acute hepatitic-type injury without cholestasis. However, “clinical,” “biochemical,” and “histological” cholestasis can be seen in varying combinations among the spectrum of AIH phenotypes.**

Clinical cholestasis is not expected in classical AIH cases. Pruritis is rarely seen, serum ALP level is normal or slightly increased, and there is no histological cholestasis. Hyperbilirubinemia is seen due to hepatocellular damage induced by pronounced inflammation in acute icteric AIH. Moreover, “histological cholestasis” (the presence of bile within hepatocytes and/or in bile canaliculi) can be seen in severe acute or acute-on-chronic AIH.^46^ As expected, biochemical, clinical, and histological findings of cholestasis are common in advanced stages of decompensated cirrhosis due to the global impairment of liver functions. So, if there are clues of clinical or biochemical cholestasis in an adult patient with AIH, the further clinical and histological analyses should be made after extrahepatic cholestasis is excluded by radiological methods. On the other hand, irrespective of cholestasis, magnetic resonance cholangiopancreatography is needed in all children with suspicion of AIH, due to high probability of existent or evolving PSC. Details in differential diagnosis and variant/overlap concepts are discussed in the relevant section.

### Is Liver Biopsy Mandatory in the Diagnosis of Autoimmune Hepatitis? Are There Any Exceptions?

Although AIH is not only a morphological diagnosis, a definitive diagnosis cannot be made without a liver biopsy, so it should be performed in suspected cases. Morphological findings detected in the liver biopsy constitute the histopathological component of the complex AIH scoring systems regarding the clinical and pathological aspects of the disease. If the history of drug-induced AIH is clear or if the patient is not suitable for immunosuppressive therapy due to its severe comorbidity, a biopsy is not required. If the percutaneous biopsy is contraindicated because of coagulopathy, transjugular biopsy is recommended.^1^

### What Is the Importance of Clinicopathological Correlation in the Diagnosis of Autoimmune Hepatitis?

**Autoimmune hepatitis is a clinicopathological diagnosis. Especially in mild, acute, drug-induced, and overlap/variant AIH cases, a prompt correlation is essential, and without correlation diagnostic errors may ensue.**

Most AIH cases can easily be diagnosed with clinicopathological correlation. A pathology report including comments on the hepatitic type of injury with interface activity, the presence of plasma cell infiltration, and the exclusion of other possible causes by clinical and laboratory findings can secure the diagnosis of AIH. Sometimes, the pathologist just reports the injury pattern as hepatitic and/or cholestatic or describes elementary lesions, without commenting on the big picture. In this setting, the habit of the clinician is to try to make an AIH diagnosis by looking at scoring systems. However, as discussed later, AIH scoring systems are not relevant and reliable for all clinical scenarios. Furthermore, the clinician may misinterpret many nonspecific elementary findings, like ductular reaction, in favor of PBC or PSC. In order to reduce diagnostic errors, the clinician should know the meaning of basic histologic elementary lesions, be aware of the need for further histological examinations such as copper stains (for chronic cholestasis) and keratin stains (CK7/CK19 for ductular reaction and duct loss), and collaborate with the pathologist (Table 1, Illustrative Case 1). If needed, a more experienced hepatopathologist opinion and longitudinal follow-up may be required to achieve a definitive diagnosis.

Regarding the scoring systems, revised 1999 and simplified 2008 criteria are usually enough for the diagnosis of classical chronic AIH (Table 2).^47,48^ Liver biopsy serves an important contribution to these diagnostic scores.^42^ In the revised system, clinical and laboratory features are scored for up to 21 points and histology for up to 5 points (a total of 10-15 points: probable AIH, >15 points: definite AIH). Regarding histology, interface hepatitis is scored as 3 points, lymphoplasmacytic infiltration as 1 point, and rosette formation as 1 point. If there are typical findings for another disease, such as steatohepatitis or destructive cholangitis, the histologic score should be decreased by 3 points. If none of the AIH histologic features is present and there are features suggesting other diseases, up to 11 points may be deducted, after which it becomes almost impossible to reach the minimum diagnostic score for AIH of 10 points. On the other hand, if scoring is applied without liver biopsy or if biopsy findings are scored incorrectly, misdiagnosis of AIH easily ensues. In the simplified system, clinical and laboratory features are scored for up to 6 points and histology for up to 2 points (a total of 6 points: probable AIH, 7-8 points: definite AIH). To these criteria, the presence of 3 histologic features is required for categorizing a case as *typical* (2 points): interface hepatitis with portal lymphocytic/lymphoplasmacytic infiltration, emperipolesis, and rosettes. If there is only chronic hepatitis without characteristic features, the case is considered as *compatible* with autoimmune hepatitis (1 point). However, emperipolesis and rosettes have limited sensitivity and specificity, and lobular inflammation and CN are not scored in both systems.^39,42^

### Are There Any Consensus-Based Histological Criteria in the Diagnosis of Autoimmune Hepatitis?

Recently, Lohse et al^49^ proposed the first consensus-based histological criteria for AIH. In the settings of chronic and acute presentations of AIH, which are usually characterized by a dominant portal or lobular inflammatory infiltrate, respectively, the consensus criteria are defined as “likely, possible or unlikely” AIH. They disregarded the old terminology of “typical, compatible, incompatible.” To their scheme, AIH is LIKELY if there is either:

- The presence of a predominantly portal lymphoplasmacytic infiltrate with more than mild interface hepatitis and/or more than mild lobular hepatitis, in the absence of histological features suggestive of another liver disease.
- The presence of a predominantly lobular hepatitis, more than mild in severity, with or without centrilobular necroinflammation and lymphoplasmacytic infiltrates, or interface hepatitis, or portal fibrosis, in the absence of histological features suggestive of another liver disease.

The report also has criteria for possible and for unlikely diagnoses of AIH.^49^ The proposed scheme seems to work in acute and mild AIH cases in addition to classical chronic AIH. However, the diagnostic criteria regarding AIH vs. DILI, NAFLD, or overlap/variant syndromes are not codified in the consensus paper, leaving them to further research. Moreover, it is not mentioned how to score the new terminology of “likely, possible and unlikely” as individual points in the histological sections of the AIH scoring systems.^50^

## Scoring Systems

### When and How Do We Use Scoring Systems in Autoimmune Hepatitis Diagnosis?

The scoring systems should not be used without a liver biopsy. Although widespread habitual use, actually routine scoring is not mandatory. These systems should be used only to support the clinical judgment in challenging cases of AIH and to define AIH cohorts for clinical studies.^1,4^ In a typical patient, the clinical evaluation is sufficient for AIH diagnosis based on liver tests, serology, histology, and exclusion of other liver diseases. If desired, the use of scoring systems may be flexible and complementary (Table 2). The revised scoring system has greater sensitivity for AIH compared to the simplified scoring system (100% vs. 95%), whereas the simplified scoring system has superior specificity (90% vs. 73%) and accuracy (92% vs. 82%), using clinical judgment as the gold standard.^4^ Keeping the caveats mentioned earlier in the section ”Histology,” the simplified scoring system is recommended for typical patients due to its simplicity and accuracy, whereas the revised scoring system is preferable for patients with complex or unusual features due to its comprehensive nature (seronegative or low titer antibodies, normal serum IgG, acute severe case, male patient, etc.). In such scenarios, the timely diagnosis of AIH can be captured by the latter, despite the score of the simplified system being lower than 6.^36^ The newly proposed histological features such as CN and updated cutoff values of serology should be incorporated into clinical judgment while applying scoring systems.^30,49^ In daily practice, the scoring systems are not recommended to use in overlap/variant syndromes.

### What Do You Mean by “Clinical Judgment as the Gold Standard” for Autoimmune Hepatitis Diagnosis?

Clinical judgment is a holistic approach to AIH diagnosis that includes the evaluation of clinical, biochemical, serological, and pathological characteristics with their *pros and cons*, and, if needed, the use of a scoring ­system appropriate for clinical AIH phenotype (Illustrative Cases 2 and 3).

## Treatment

Autoimmune hepatitis cannot be cured with the current therapies, so the main goal is to achieve remission. Although remission can be obtained in the majority of cases, the treatment process is difficult and problematic. The duration of treatment is long, that is, induction of remission takes months, and maintenance of remission takes years. In addition, many factors such as, patient adherence, side effects of drugs, insufficient adjustment of immunosuppressive therapy due to inexperience, etc. may impede remission. Finally, when attempting to discontinue treatment even after a durable remission, relapse usually occurs, so AIH treatment is life-long in most cases.^1,4,27,28,36^

## Indications

### In Which AIH Presentations Is the Immunosuppressive Treatment Indicated and in Which It Is Not?

**All the clinical forms of AIH including patients with acute hepatitis-chronic hepatitis-cirrhosis are candidates for treatment if the disease is active. When making a treatment decision in mild disease, extreme forms (ALF, ACLF, decompensated cirrhosis), overlap syndrome, and in patients with the concurrent disease (NAFLD, viral hepatitis, SLE, etc.), the treatment decision should be individualized.**

According to a number of randomized trials in the 1960s and 1970s, steroid therapy significantly improved survival in patients with severe AIH defined by ALT, AST >10× upper limit of normal (ULN), or >5×ULN plus IgG >2×ULN, or histological features of bridging or multilobular necrosis (5- and 10-year survival of 50% and 10% in untreated patients vs. 10-year survival of 90% in treated patients).^51,52^ Following trials have established the role of azathioprine (AZA) as steroid-sparing agent and in the maintenance of remission.^1,4^ In the long-term studies conducted thereafter, it was shown that patients with less severe disease (i.e., interface hepatitis on histology and milder elevation of laboratory tests) and even mild asymptomatic cases, if not treated, had a higher risk of cirrhosis and mortality compared to the normal population.^27,53^ Therefore, according to the guidelines, the treatment indications included the most severe patients described earlier in the 2002 and 2010 AASLD, while the 2011 BSG (British Society of Gastroenterology) expanded to include less severe patients and finally, all patients with active liver inflammation (mHAI >3 on histology *with or without* abnormal serum transaminases, IgG levels, or the presence or absence of symptoms) were accepted as candidates for treatment in the 2015 EASL and 2019 AASLD.^1,4,27,51,52^

**Figure 4.**
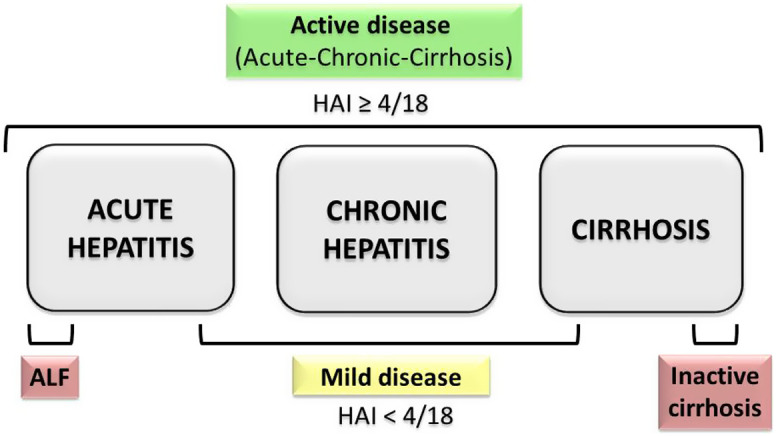
Treatment is indicated in active disease (green box), in selected cases of mild disease (yellow box), and it is not recommended in extremes (red boxes).

Generally, all AIH groups including patients with acute hepatitis, chronic hepatitis, and cirrhosis are candidates for treatment if the disease is active (Figure 4). When making a treatment decision in mild disease (ALT <3ULN; mHAI ≤3 and no advanced fibrosis), the age of the patient, co-morbidity, serology, and preferences should be taken into account.^1^

In extremes such as decompensated cirrhosis or ALF, great caution is required. Decompensated cirrhosis may deserve low-dose prednisolone therapy if there are laboratory or biopsy findings of active inflammation, but infections should be excluded throughout the therapy.^54^ Treatment is not indicated in inactive/burnt-out cirrhosis. Moreover, in this setting, a confident diagnosis of AIH is difficult because burnt-out non-alcoholic steatohepatitis (NASH) is far more common than AIH, and cirrhotic NASH patients may have positive non-specific serology and elevated IgG.^55^ Cases with encephalopathy (ALF) should be evaluated directly for liver transplantation, and if steroids are to be tried in earlier stages, it should be done in a transplantation center.

In overlap syndrome, and in patients with the concurrent disease (NAFLD, viral hepatitis, SLE, etc.), the treatment decision should be made case by case.

### Pre-Treatment Evaluation

Most patients with AIH need life-long immunosuppression.^1^ Patients should be well informed about possible side effects that may develop during induction or maintenance. If available, screening patients with AIH for thiopurine methyl transferase (TPMT) activity prior to initiating treatment with AZA is recommended. Screening viral serology including HBsAg, anti-HBc, and anti-HCV are routine parts of the diagnostic process in AIH. Detection, prevention, and treatment of HBV reactivation are important during immunosuppressive treatment (IST) which is planned according to the current guidelines.^4^ Vaccination status should be reviewed and vaccines should be administered to all susceptible patients with AIH according to the age-specific guidelines. IST increases the risk of bacterial and fungal infections, especially in severe cases with ACLF, ALF, or cirrhosis, but mild neutrophilia due to steroid use is common and not regarded as a sign of infection in the absence of supportive clinical and laboratory findings. Corticosteroids negatively affect glucose regulation in patients with concomitant diabetes mellitus and can worsen underlying fatty liver disease. The optimal initial doses and type of corticosteroids should be decided carefully and these patients should be managed together with an endocrinologist and dietician. Baseline bone mineral density should be performed before corticosteroid therapy due to the risk of osteoporosis. Vitamin D and calcium supplements should be given to patients who have bone disease. Patients with AIH are also associated with a high risk of depression and anxiety. These patients need psychiatric support which can also increase treatment compliance and may reduce the risk of rejection perception against AIH diagnosis.^1^ Fertility and pregnancy are important issues because some of AIH patients are first diagnosed while evaluated for infertility. All AIH patients who are diagnosed at reproductive age should be acknowledged about the optimal time for conception and possible maternal and fetal outcomes.^56^

### Treatment Targets and Responses

**Figure 5.**
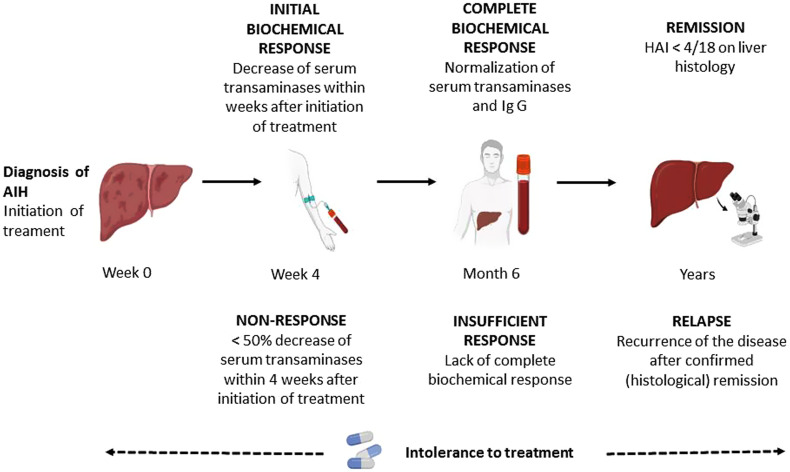
The definition of treatment responses in autoimmune hepatitis (adopted from the consensus report,^57^ initial response and relapse were added for didactic purposes, positive responses are shown in the upper set and negative responses are shown in the lower set).

The main aim of first-line therapy is to control hepatic inflammation at the lowest risk of drug-induced complication.^4^ The non-invasive measure showing that the histological inflammation subsides is the normalization of serum transaminases and IgG levels. With treatment, first transaminases, then IgG, and finally histological findings improve. According to the recently published consensus report,^57^ treatment responses are defined as given in Figure 5.

**Complete biochemical response: **Complete biochemical response (CBR) is defined as the normalization of serum aminotransferases and IgG levels within 6 months after treatment.

**Insufficient response: **Insufficient response (IR) is defined as the inability to obtain a complete biochemical response.

**Remission: **Remission is described as liver histology with an HAI <4/18 or equivalent.

**Treatment intolerance:** The occurrence of treatment-related adverse events, potentially leading to drug discontinuation, as assessed by the treating physician.

**Non-response:** Non-response group is defined as a less than 50% decrease from baseline in serum transaminases at week 4 of the treatment.

## First-Line Treatments

### How Is the Development of AIH Treatment According to the Guidelines?

High-dose prednisone (60 mg/day) monotherapy or low-dose prednisone (30 mg/day) plus fixed-dose AZA (50 mg/day) combination therapy was first recommended in 2002 and subsequently in 2010 AASLD guidelines.^51,52^ These regimes had similar beneficial effects but the combination regime was associated with fewer side effects than prednisolone alone. In BSG 2011 guideline, prednisolone 30 mg/day plus AZA 1 mg/kg/day was recommended, while EASL 2015 guideline proposed combined treatment with higher doses of prednisolone 0.5-1 mg/kg/day and AZA 50 mg/day followed by 1-2 mg/kg/day and, finally, prednisolone 20-40 mg/day plus AZA 50-150 mg/d was recommended in the AASLD 2019 guideline.^1,4,27^ In a recently published multicenter European study, low-dose steroid therapy was shown to be as effective as high-dose therapy.^58^ Moreover, in the community-based studies, and to the evaluation of expert opinions, it was shown that very different treatment regimens were applied in daily clinical practice.^2,3^ Although, there is still some debate regarding the optimal dosage of these drugs, the basic philosophy of the AIH treatment process is the need for an individualized, response-guided, stable, and long-term process.^1^

### What Is a Typical Treatment Course and the First-Line Treatment Options for a Patient with Autoimmune Hepatitis?

**A typical treatment course consists of 4 phases including remission induction, remission maintenance, treatment cessation trial, and relapse. Steroids are the drug of choice for remission induction, and AZA is the drug of choice for the maintenance of remission. Initial steroid choice and drug doses should be determined by considering the disease activity, the patient characteristics, and drug-related side effects altogether. Prednisolone followed by AZA is usually administered during a remission induction phase as first-line treatment. Azathioprine requires several weeks to achieve efficacy. Budesonide can be a choice in mild-moderate non-cirrhotic cases but not in acute severe presentation or cirrhosis.**

**Figure 6.**
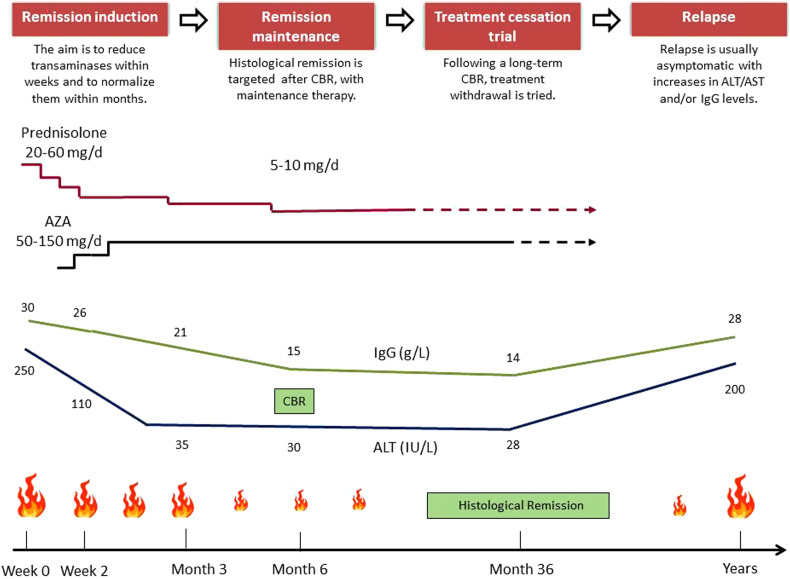
The phases of a typical treatment course in a classical responder patient with AIH. The first-line treatment options, serum ALT, IgG and histological inflammation is illustrated on the longitudinal time scale. AIH, autoimmune hepatitis; ALT, alanine aminotransferase; CBR, complete biochemical response; IgG, immunoglobulins.

**Figure 7.**
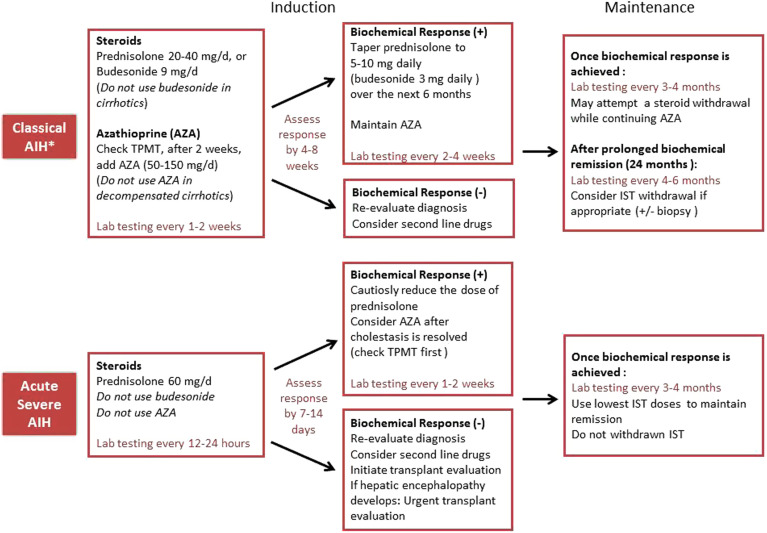
The first-line treatment of AIH. Source: Adopted from AASLD 2019 guideline. *Classical AIH includes chronic or acute cases with mild-to-moderate severity and cirrhotic cases. AIH, autoimmune hepatitis; IST, immunosuppressive therapy; TPMT, thiopurine methyl transferase.

1. **Remission induction:** Combination therapy (steroid followed by AZA) or less commonly monotherapy (steroid) is administered during a remission induction phase (Figures 6 and 7). At this phase, the aim is to reduce transaminases within weeks (initial biochemical response) and to normalize them within months (biochemical remission). Initial steroid type and dosage are determined by the disease activity and the patient characteristics, ranging from 20-60 mg/day or <0.5-1 mg/kg/day for prednisolone and 9 mg/day for budesonide (Figure 7). The decided steroid tapering scheme is explained to the patient. Azathioprine is added 2 weeks later, after confirming steroid responsiveness, excluding the rare possibility of AZA-induced hepatitis by checking declining serum transaminases and total bilirubin <6 mg/dL and performing TPMT test if available.^1,4,27,28,52^ The initial AZA dosage is 50 mg/day and increased depending on toxicity and response up until a maintenance dose of 1-2 mg/kg. Meanwhile, the prednisolone is firstly tapered gradually to a level of 10 or 20 mg/day, as serum transaminases fall, as a reduction of 5-10 mg per weekly intervals. (This period takes a few months according to the selected tapering scheme of EASL or AASLD. In prednisolone followed by AZA-combined schemes, the initial dose of prednisolone in the range of 20-60 mg is reduced to 10 mg, while the initial dose of 60 mg in the prednisolone monotherapy scheme is reduced to 20 mg.) Further tapering of prednisolone below 10 or 20 mg/day should be done after confirming repeated normal values of transaminases and should be slow as a reduction of 2.5 mg per 3-month intervals till 5-10 mg/day, aiming complete withdrawal at a point of subsequent “remission maintenance phase” (Table 3, Figure 6).
  Steroid tapering differs according to the treatment scheme chosen previously, the main point is that if there is an increase in transaminases during steroid tapering, tapering should be done more slowly. In such circumstances, it may be necessary to gradually increase the dose of AZA to maintain the decrease in serum transaminases. According to the consensus report,^57^ the CBR target is achieved in an average of 6 months but earlier in some patients and later in others. So, the adjustment of immunosuppression should be individualized and response guided.^1^
  Mycophenolate mofetil has also been proposed as an alternative first-line option and seemed to be effective as much as or even more than AZA,^28,59^ but the current AASLD guideline concludes against its first-line use due to limited data.^4^ The cost and safety issues regarding pregnancy should be taken into consideration while planning MMF usage.
2. **Remission maintenance:** When biochemical remission is achieved in a mean of 6 months (i.e., ALT and IgG normalization, IgG may be delayed), histological remission is targeted with remission maintenance therapy. An already tapered lower dose of prednisolone (5-10 mg daily), with an adjusted dose of AZA, usually maintains biochemical remission. Prednisolone may then be discontinued completely in a slow manner, leaving the patient on only AZA.^4^ During a typical treatment course, prednisolone is used for an average of 6 month-1.5 years and AZA for 3 years.
3. **Treatment cessation trial: **Although drug withdrawal and achievement of long-term treatment-free remission of AIH are possible in a minority of patients,^60^ it is desirable for many reasons, including patient choice, side effects, and potentially increased cancer risk due to immunosuppressive treatment (IST).^4,61^ The attempt of treatment withdrawal should be considered if serum aminotransferase and IgG levels have been persistently normal (i.e., stable remission while on AZA monotherapy) for at least 2 years, usually after 3 years of therapy.^1,4^ Therefore, if the patient is still under combined therapy, steroid should be tapered and discontinued first, if possible during the second half of the first year of treatment, while maintaining remission on AZA 1-2 mg/kg for another 2 years at least. Flares of AIH activity during steroid tapering or AZA maintenance require increased doses of immunosuppression and preclude complete drug withdrawal.^1^ Finally, AZA monotherapy is tapered 25 mg every 2 weeks or even slower and discontinued.^61^
  Liver biopsy before treatment cessation trial is optional in adults according to the current guidelines.^1,4^ Due to its invasiveness and sampling errors and due to the increasing evidence for biochemical remission as a reliable predictive marker, it has become less important in the assessment of remission.^62,63^ Many prefer complete biochemical remission and serial Fibroscan measures to define disease control.^61^ Patients with ALT levels in the lower half of the normal range and IgG levels <12 g/L have a higher chance of successful treatment withdrawal than patients with values in the upper range of normal.^62^ Although the liver biopsy has limited value in predicting AIH relapse, it may be necessary to detect ongoing inflammation or fibrosis progression, if there is doubt that biochemical parameters are reliable, or to distinguish remaining AIH activity from other causes of elevated liver enzymes (drug toxicity, associated NASH, etc.).^61,63^
  Treatment withdrawal should be avoided during puberty or in patients with the acute severe presentation, and it requires great caution in patients with childhood-onset or type 2 AIH.^63^ Patients with advanced cirrhosis should be advised against drug withdrawal since a flare might cause hepatic decompensation. Previous relapsers and SLA/LP-positive patients have also a higher risk of relapse with the need of permanent immunosuppression.^1,64^
4. **Relapse: **Relapse occurs in 50%-80% of adults after drug withdrawal. Although many factors have been proposed, the principal factors for relapse are the duration and completeness of inactive disease before treatment withdrawal.^62^ It is presented as increases in serum transaminases and/or IgG level and occurs usually in the first 3 months. Relapse monitoring with laboratory testing is required regularly in the first year and then at annual intervals for lifelong. Relapse requires reuse of the original treatment until biochemical remission and subsequently a long-term maintenance regimen usually with AZA at a dose of up to 2 mg/kg/day.^1,4^ In reality, it is a tiny proportion of patients who can be safely withdrawn from therapy altogether. Even patients who have been stable for years can have a flare with an acute event (spontaneous activation, post-viral infection, etc.). So, it is not unacceptable to consider keeping on indefinite immunosuppression.

### In Extremes of Autoimmune Hepatitis Spectrum Such as Acute Severe Hepatitis/Acute Liver Failure and Acute on Chronic Liver Failure, Which Issues Should Be Taken into Consideration in Making the Treatment Decision?

**The most important point of treatment in acute severe AIH is early diagnosis! The sooner the steroid therapy is started, the higher the chance of success. Active search for infection, appropriate management of developing OF(s), and dynamic evaluation of the need for transplantation are required to investigate during treatment. Evaluation of response to steroid therapy should be interpreted within 2 weeks or even earlier. Cases with encephalopathy (ALF) should be evaluated directly for liver transplantation, and if steroids are to be tried, it should be done in a transplantation center. The place of immunosuppressive treatment is not clear for AIH-ACLF. To limited data, it may be effective in the earlier phase of ACLF (MELD <24, no active infection or organ failure). Immunosuppressive treatment should not be started in more severe patients with ACLF, and it should be withheld if the patient is currently using it until stabilization.**

**In ASH/ALF and ACLF, falling transaminases are not a marker for hepatic recovery in the face of rising INR and bilirubin, as they may fall due to a loss of functional liver mass. Response assessment in the ALF-AIH group should be made with INR, bilirubin, MELD score, Survival and Prognostic Factors for Acute Severe Autoimmune Hepatitis (SURFASA) score, and clinical findings and not only with transaminases and IgG as in classical chronic AIH. Similarly, within the ACLF-AIH cases, the EASL or APASL scoring system should be used in a dynamic manner.**

In the presentation of acute AIH, many possible scenarios exist, namely truly acute or acute-on-chronic in the setting of chronic hepatitis or cirrhosis. The definitions of the classification in acute cases have only been made recently (Figure 1), without an international consensus, and many controversies exist especially in ACLF. Diagnostic issues regarding acute AIH were discussed previously. It is estimated that around 50% of acute severe AIH cases will progress to ALF, while the percentage of ACLF is unknown. Steroid responses ranging from 20% to 100% have been reported in previous AIH-ASH studies, probably due to the inclusion of diverse acute presentations and different treatment regimens.^4^ The investigators concluded that treatment success is related to timing (sooner vs later), stage (acute vs. subacute/late-onset), severity (INR <2 vs. >4, MELD <27 vs. >40, and the absence of encephalopathy vs. higher degrees of encephalopathy).^5,65^ The rate of death and transplantation increases significantly in the severe scenarios. Steroid dosing and the route also varied among the institutions. Our preference is prednisolone, at a dose of 60 mg or higher per day oral or i.v. depending on severity. Tacrolimus may be effective in some cases of steroid non-responders during the course of transplant evaluation.^66^

Protracted steroid therapy may cause delay or loss of chance in the transplantation process due to developing infection. The increased risk of infection is related to the duration of steroid use (mostly >2 weeks), and AIH severity itself, not to the steroid dose. So, if there is no improvement in INR, bilirubin or MELD after 7-14 days of steroid therapy, continuing them may be futile and patients should be assessed for LT immediately.^4^ In advanced cases with encephalopathy, earlier assessment on day 3 of steroid therapy may be better.^67^

Only a few studies specifically examined AIH-ACLF, and mainly those with ACLF according to the APASL concept were included.^68,69^ The severe ACLF group with infection or extrahepatic OF(s) was excluded and for those steroids that were not given, only supportive therapy or liver transplantation was planned. In the patients of AIH-ACLF from the APASL cohort (21% with cirrhosis), a transplant-free survival rates of 75% was obtained and suggested early stratification to steroid therapy (MELD <27) or liver transplantation (MELD >27, hepatic encephalopathy in ≥F3).^68^ A more recent report included 29 patients of AIH-ACLF (all with cirrhosis), a transplant-free survival rate of 55% was obtained, and a MELD score < 24 had the best predictive value for survival^69^ (Illustrative Cases 4, 5).

### Insufficient Response and Non-Response

Although AIH is generally sensitive to IST, complete response is not a rule, a significant proportion of patients are grouped as insufficient responders (13%) and non-responders (7%). This actually indicates ongoing histological inflammation regardless of the arbitrary subdefinitions. In addition to ongoing inflammation and activation of AIH itself, many treatment-related and diagnosis-related factors may complicate the evolution of the disease course.^52^

### Insufficient Response

**Figure 8.**
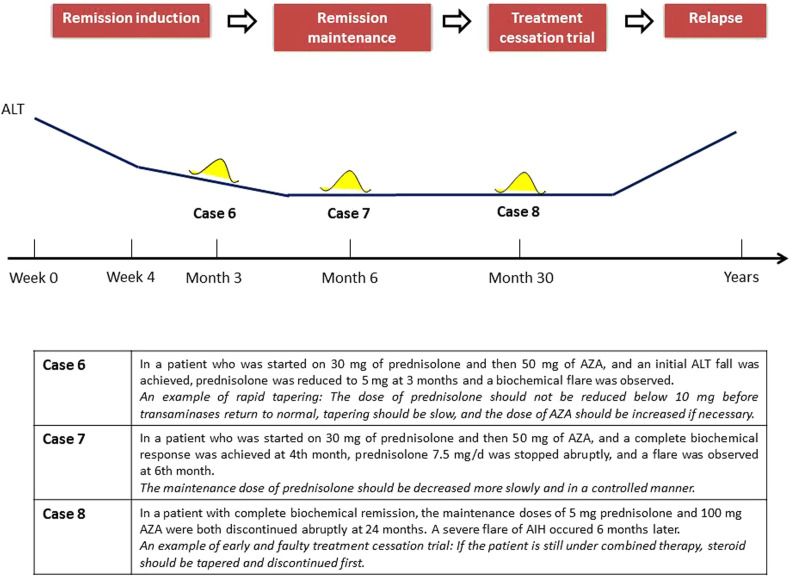
Some illustrative examples of insufficient response spectrum are shown on the longitudinal time scale of autoimmune hepatitis.

The consensus report stated that any response other than normalization of transaminases and IgG levels, no later than 6 months after initiation of treatment, should be classified as an IR, after standard therapy has been applied and adherence has been proven.^57^ This definition primarily refers to “true” IR due to persistent inflammation of AIH itself and requires planning of second- and third-line therapy. The consensus noted that ALT or AST elevation due to an obvious alternative etiology will preclude the achievement of a complete biochemical response and will be marked as an IR. Moreover, relapse during maintenance therapy may also be considered a version of the IR, usually due to the lowering of maintenance therapy down to a level lower than required in the individual patient or due to non-adherence.^63^ So, keeping in mind the longitudinal time course of AIH treatment, this wider definition allows a comprehensive listing of the possible causes and differential diagnosis of IR spectrum and its specific management actions (Table 4, Illustrative Cases 6, 7, and 8 in Figure 8).

The various causes of IR can be examined in 3 groups which are interrelated:

1. **Disease-related causes (“true” IR due to intrinsic resistance, activation, or disease progression of AIH itself):** With the initial standard treatment course, biochemical remission in 80% and histological remission in 60% of patients are achieved in 2 years. Notwithstanding this high initial remission rate, long-term management of AIH remains suboptimal due to frequent relapses after discontinuation of treatment and sometimes despite continuing treatment. Liver fibrosis may still progress and de novo cirrhosis develops in 18% of patients under treatment according to the old response criteria (AST <2 ULN). This is probably because of incomplete suppression of liver inflammation even in presumed responders defined by old criteria.^61^ While still not perfect, detection of disease activation and progression can be done effectively by using new biochemical criteria of ALT and IgG normalization.
2. **Treatment-related causes (insufficient immunosuppression):** The main causes are patient inadherence to drugs and stopping the therapy due to side effects of drugs. The other issues are related to mistakes in treatment course such as inadequate dosing adjustment according to response (i.e., low drug dosages and rapid tapering of steroid), wrong drug selection for the particular case, and premature stopping of treatment before an established response.
3. **Diagnosis-related causes (incorrect AIH diagnosis and other added/variant diagnoses):** Incorrect AIH diagnosis may arise from clinical heterogeneity, some weakness of the characteristic serological and histological features such as technical reasons, sampling errors and operator inexperience, and imperfect accuracy of the AIH scoring systems across the non-classical AIH phenotypes. Therefore, to diagnose AIH correctly, other hepatobiliary diseases must be ruled out through appropriate diagnostic algorithms. Moreover, many disorders can be added to AIH during the disease process, most commonly DILI, NASH, viral hepatitis, celiac disease, and various rheumatologic and immunologic disorders.^1,4^ The differential diagnosis may require appropriate noninvasive testing and repeat liver biopsy. The details of AIH and PBC and PSC overlaps are given in the relevant section. Of course, in the appropriate clinical setting, the other non-specific common conditions such as infections, decompensated heart failure, biliary stones, and malignancies should also be excluded as an intercurrent cause of elevated liver tests and/or clinical deterioration.

### Non-response

Non-response is defined as “<50% decrease of serum transaminase levels within 4 weeks after initiation of treatment.”^57^ Non-response is not well-studied in AIH. Considering the steroid responsiveness of AIH, non-response should question the diagnosis and adherence to treatment at first. Non-responders with immediate severity are treated as acute severe AIH. In non-responders without immediate severity, after confirmation of diagnosis and adherence, the management approach includes intensification of standard treatment by higher doses of prednisolone and AZA, determination of AZA metabolites, and applying other second- and third-line options by expert advice.^1,27^

## Second- and Third-Line Treatments

**Figure 9.**
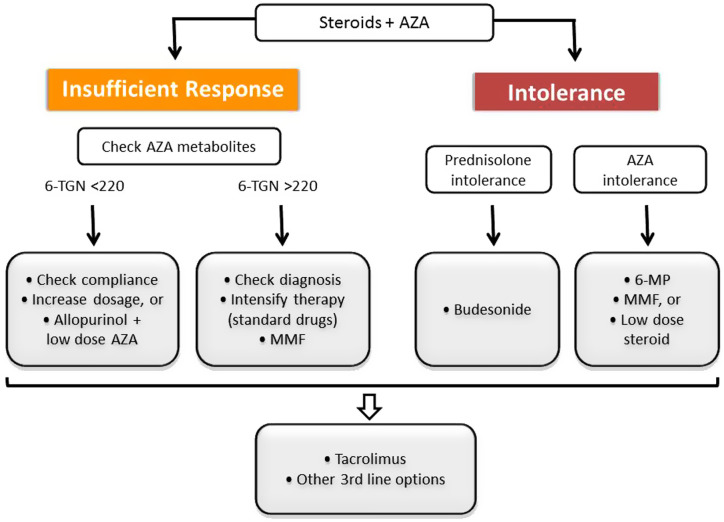
The second- and third-line treatment options in autoimmune hepatitis.

Up to two-thirds of patients with the IR and intolerance to steroid and AZA treatment can be managed with second- and third-line treatments (Figure 9). European Reference Network on Rare Hepatological Diseases (ERN RARE-LIVER) position statement recommendations are summarized here.^63^

Second-line options for the IR group include intensification of standard therapy by measuring AZA metabolites and MMF. The active metabolite of AZA is 6-TGN. If the level of 6-TGN is low, it indicates poor patient compliance or a low effective dose of AZA. If the level of 6-TGN is high but AZA is not effective, there is doubt in the diagnosis or intrinsic resistance to treatment. The options are listed in Figure 9. Since the measurement of AZA metabolites is not performed in most centers, MMF is the most widely used second line drug with 0.5-2.0 g/daily doses.^4,70^ The options in the intolerance group are MMF, 6-mercaptopurine or steroid monotherapy for AZA intolerance, and budesonide for prednisolone intolerance (Figure 9).

Third-line options include tacrolimus, cyclosporine, everolimus, rituximab, infliximab, methotrexate, sirolimus, and belimumab, but experience regarding usage of these agents is limited with small cohort sizes or case series.^63^ Most experience has been reported with tacrolimus, with a dose of 0.1 mg/kg twice daily, aiming for trough levels of 6-8 ng/mL initially and 3-5 ng/mL on remission.^71^ For intolerant patients, a well-tolerated single third-line drug will probably be sufficient, but, for cases with IR under first- and second-line therapy, double or even triple immunosuppression may frequently be needed. Planning of third-line treatments in specialist centers and sharing the results in databases such as ERN RARE-LIVER are strongly recommended for a better understanding of the efficacy and risks of these options and for standardization of treatments in the future.^63^

## Treatment Side Effects and Management

Corticosteroids have several side effects (up to 80% after 2 years) in AIH including, cosmetic changes (weight gain, facial rounding, and hirsutism), diabetes mellitus, emotional instability or psychosis, opportunistic infections, ophthalmological problems, hypertension, osteoporosis, and myopathy (Table 5, Illustrative Case 9). These side effects are mainly observed at doses >20 mg/day, but a recent study showed that even low-dose prednisolone (1-10 mg/day) was associated with an increased risk of bone fractures, development of cataracts, and diabetes in patients with AIH.^72^ Up to 25% of patients with AIH develop side effects from AZA and some of them require withdrawal of the drug (Illustrative Case 10). A recent international multicenter study showed that 15% of patients discontinued AZA therapy in the first year of treatment.^73^ Gastrointestinal toxicity (nausea and emesis), cytopenia, liver injury, pancreatitis, arthralgias, and fever and skin rash were the main causes that led to AZA discontinuation. Azathioprine-related side effect rates were not different in early and delayed starters and in cirrhotic and non-cirrhotic AIH patients.^73^

## Liver Transplantation in Autoimmune Hepatitis

The indications for LT in AIH are acute/subacute or chronic liver failure or hepatocellular carcinoma. The outcomes are generally well satisfactory, with a 5- and 10-year overall survival of 86% and 73%, respectively.^1,4^

The frequency of recurrent AIH ranges from 10% to 68%, which increases with time after LT. It is a cause of graft dysfunction associated with reduced graft and patient survival. De novo AIH is a clinical entity resembling AIH and develops in patients transplanted for etiologies of liver diseases other than AIH. It has been estimated at 1%-2% of adult recipients. The recurrent and de novo AIH can progress to cirrhosis, graft loss, and re-transplantation. The clinicopathological manifestations and treatment approach of recurrent AIH and de novo AIH are similar to those of original AIH, depending on the disease presentation.^27,28^

## Other Clinical Variants and Important Differential Diagnoses

### Overlap/Variant Syndromes

The overlap syndromes continue to be controversial after a decade has passed since the release of the only consensus report from IAIHG.^74^ It has many diagnostic and therapeutic implications despite many unresolved challenges. In clinical practice, overlap syndromes should be considered for patients who are either non-responders to standard therapy or with unusual clinical features.

### Can We Describe “Overlap Syndrome” as a True-Distinct-Another Autoimmune Liver Disease Similar to Autoimmune Hepatitis, Primary Biliary Cholangitis, or Primary Sclerosing Cholangitis?

**No. Heterogeneous nature of autoimmune liver diseases (AIH, PBC, PSC), the absence of pathognomonic criterion for any of them such as serology, and the abuse of scoring systems may lead to many artificial “overlap syndrome” diagnoses. Mostly, these conditions are *variants of the classical diseases* and a careful evaluation can usually reveal the predominant disease.**

**Figure 10.**
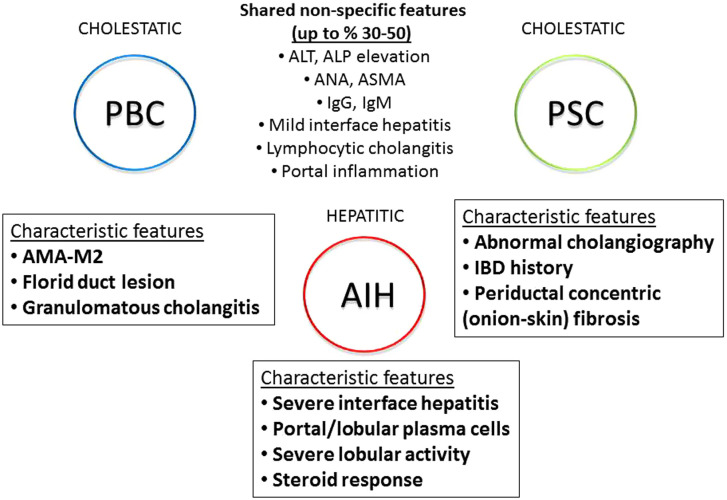
Heterogenous nature of autoimmune liver diseases.

**Figure 11.**
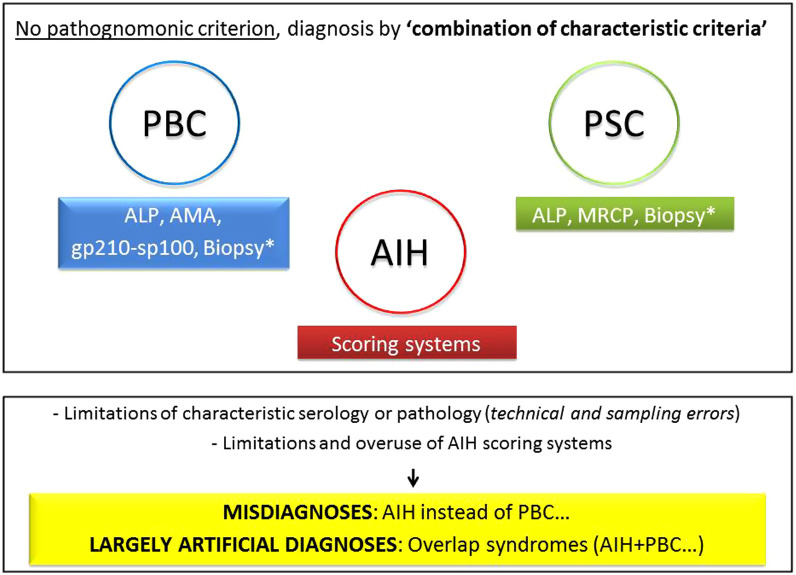
The diagnostic criteria of classical autoimmune liver diseases (upper box) and the origins of variant/overlap phenotypes (lower box). (*Liver biopsy for PBC: Serology negative or marked hepatitic features. *Liver biopsy for PSC: Small-duct involvement or marked hepatitic features.)

Autoimmune liver diseases are heterogeneous in nature.^74^ Autoimmune hepatitis is mainly a hepatitic disorder, while PBC and PSC are grouped as chronic cholestatic diseases. Despite this generalization, some cholestatic findings in AIH and some hepatitic findings in PBC and PSC are frequently observed (Figure 10). Primary biliary cholangitis and PSC may have features of AIH such as elevated serum aminotransferases, ANA-ASMA positivity, and interface hepatitis on histology, while some degree of serum ALP elevation, seropositivity for anti-mitochondrial antigen (AMA), and lymphocytic cholangitis on histology can be detected in AIH. Indeed, in clinical practice, it is possible to encounter any combination of overlap features shown in Figure 10 in a particular case. Among these features, AMA-M2 for PBC, strictures for PSC, and severe interface hepatitis rich in plasma cells and steroid responsiveness for AIH are regarded as relatively more specific and stronger criteria than the others.^22,23,74-77^ But there is no “pathognomonic” single criterion for the diagnosis of autoimmune liver diseases (such as HBsAg/HBV-DNA in hepatitis B), therefore, the diagnosis of individual disease of AIH, PBC, or PSC is based on a “combination of characteristic criteria,” after ruling out other possible causes (Figure 11). Although, they are generally differentiated easily and a correct diagnosis is obtained, overlapping features sometimes prevent a clear conclusion. In addition, even the characteristic serological and histological features are prone to some weakness due to technical reasons like sampling errors, and due to false interpretation of findings by inexperienced clinicians or pathologists. In fact, the concept of “overlap syndrome” has first been introduced upon the “AIH” overdiagnosis in 20% of patients with PBC and PSC, when the original AIH scoring system had been applied to define hepatitic characteristics in PBC and PSC patients.^20,21^ (Illustrative Cases 11, 12).

So, as stated in the consensus report,^74^ there is *no overlap as simultaneous 2 separate diseases*, there are concurrent overlap features accompanying the classical disease such as PBC, PSC, or AIH, these conditions should be described as *variants of the classical diseases*, and a careful evaluation can usually reveal the predominant disease (Figure 11). Overlap features usually occur as AIH features and added to the PBC or PSC, *simultaneously* or *sequentially*. Theoretically, the co-occurrence of PBC, PSC, or AIH—which are already rare diseases—in the same person at the same time may be an extremely rare situation.^78^ Although the consensus report did not suggest any scoring system for overlap diagnosis, modified Paris criteria by EASL are currently the most commonly used tool for diagnosing PBC/AIH overlap in which the inclusion of moderate-to-severe interface hepatitis was mandatory.^79,80^ In childhood, the term “autoimmune sclerosing cholangitis (ASC)” is used for PSC/AIH overlap, assuming it as a distinct disease process. Recently, the ESPGHAN committee proposed a scoring system for ASC in the pediatric group which needs validation.^81^ However, in a current report based on extensive analysis of published PSC/AIH cases, it is stated that ASC and PSC/AIH-overlap are not distinct entities, but they represent “inflammatory” phases of PSC manifesting earlier in the disease course, which evolves into a more classical PSC phenotype in later life.^82^

### What Are the Main Clinical Consequences of Inaccurate Interpretation of Variant Features Among Autoimmune Liver Diseases?

**Overdiagnosis leads to unnecessary, harmful, or futile treatments, while false diagnosis or undertreatment carries the risk of disease progression.**

Misinterpretation of overlapping hepatitic features exposes the PBC or PSC patient to the detrimental effects of long-term IST, while ursodeoxycholic acid (UDCA) is unnecessarily added to the treatment by looking at the signs of mild cholestasis in a patient with AIH. The most dramatic scenario was reported by^83^ a patient with “acute severe AIH” diagnosed inaccurately as PBC, because of the false positivity of AMA and misinterpretation of nonspecific biliary features in the biopsy. The patient underwent liver transplantation due to lack of early steroid therapy (Illustrative Case 13).

### How Can We Minimize the Over-Diagnosis of Overlap Syndromes?

**The evaluation of clinical clues and extended serological work-up with a careful and detailed histological analysis are required to arrive at an accurate diagnosis.**

Clinically, PBC usually shows an indolent course without severe flares or acute liver failure, while AIH and PSC have a more heterogeneous and exacerbating course. Primary biliary cholangitis is characterized by “chronic incomplete cholestasis” for years because of the focal-segmentary involvement of the biliary tract.^37,43^ In contrast to diffuse/near diffuse cholestasis seen in DILI, viral causes, and mechanical large duct obstruction, there is no clinical jaundice or histological cholestasis but only an increase in the ALP level in the earlier stages of PBC. Obstructive etiologies are characterized by acute or subacute episodes of clinical jaundice, histological cholestasis, or even acute cholangitis. Severe acute presentations of AIH and obstructive episodes of PSC may behave in a similar pattern. However, clinical jaundice in PBC is remarkable only in patients with severe ductopenia or late cirrhosis or in the presence of concomitant causes.^41^

To increase the rate of serological diagnosis, PBC-specific ANAs (sp100 and gp210), soluble liver antigens (SLA), LKM-1, and p-ANCA should be added to ANA, ASMA, AMA measurements, technical problems should be resolved, and tests should be repeated if necessary. For instance, with the complementary use of IF, ELISA and IB, 80% positivity of AMA/M2 can be increased up to 90%-95%.^22^ Moreover, with the addition of PBC-specific ANAs to the serological work-up, only less than 5% of PBC cases remains seronegative. Similar to PBC-specific ANAs, SLA in AIH has a low sensitivity of around 30%, but specificity is excellent.^76^

**Figure 12.**
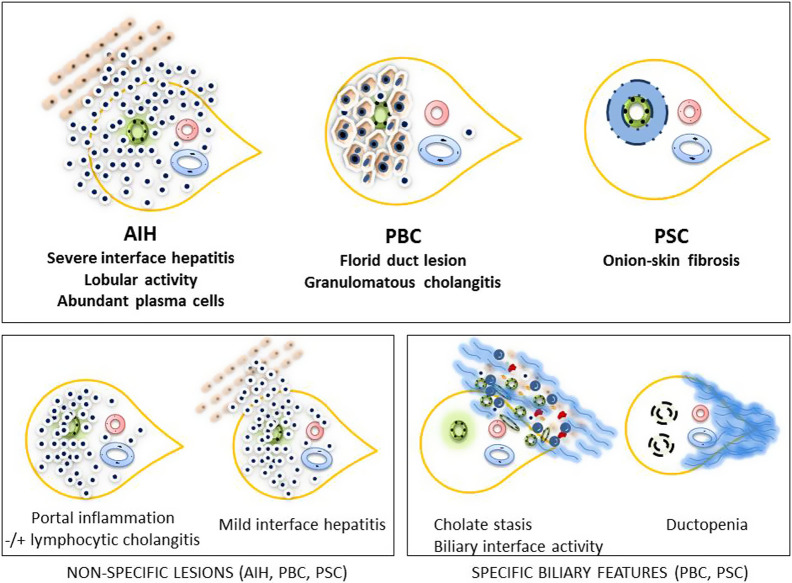
The illustration of typical (upper box) and compatible (lower boxes) histological features of classical autoimmune liver diseases.

Histologically, the main targets of inflammation are hepatocytes in AIH and bile ducts in both PBC and PSC. While AIH is characterized by interface hepatitis which is the spread of the inflammation from the portal area into the lobule, PBC affects the small-sized interlobular bile ducts in the form of “chronic, non-suppurative destructive cholangitis” and PSC causes “fibrosing cholangitis” mainly involving larger ducts (Figure 12).^37,84,85^ While bile duct damage defined as “lymphocytic cholangitis” is a nonspecific early finding with the potential for progressive destruction and loss of the bile duct (ductopenia) in PBC and PSC, it can also be seen in AIH as a “bystander” injury without progressing into a chronic biliary process. In PBC, when the portal “lymphohistiocytic“ inflammation is marked and there is “loosely formed or fully established epithelioid granulomatous cholangitis,” the term “florid duct lesion” is used which is almost virtually pathognomonic for PBC. In the case of PSC, onion-skin obliterating fibrosis in the form of “periductal concentric fibrosis” is typical. In both biliary diseases, portal changes progress into periportal areas mostly inducing ductular proliferation with accompanying “biliary interface activity” and fibrosis named “ductular reaction” resulting with “cholate stasis.” Cholate stasis is the most specific component of chronic progressive ductopenic biliary diseases meaning hepatocyte injury due to the accumulation of toxic bile salts and hepatocellular copper deposition, which both can be detected histologically. Both copper deposition and hepatocyte injury are initially seen at the periportal region (zone 1) resulting in the appearance of “periportal halo.” Hepatocyte injury is seen as the rarefaction of the cytoplasm and increase in the size of the hepatocytes causing the appearance of “ballooning degeneration” which is named as “feathery degeneration” also in the case of accompanying bile salt accumulation. Degenerated keratin filaments can be seen as “Mallory-Denk bodies” in the cytoplasm of ballooned hepatocytes. On the other hand, “lymphocytic interface activity’’ (interface hepatitis), which is a frequent histologic feature of AIH, may also be present focally in PBC or PSC, but hepatocyte necrosis is unusual and the severity of infiltration is usually milder than AIH. The presence of granulomatous cholangitis together with the prominent interface and lobular activity should alert the pathologist about overlap syndromes, and in these circumstances, evaluation of histopathologic findings by clinical data is needed. In advanced stages of PBC or PSC, bridging fibrosis in the porto-portal pattern and ductopenia become more apparent and, finally, a biliary type of cirrhosis develops.

If the clinician is aware of the meaning of the elementary findings described previously, clinical-pathological cooperation may become easier. Among these findings, portal inflammation, interface hepatitis, lymphocytic cholangitis, fibrosis, and ductular proliferation are nonspecific lesions that can be seen in chronic hepatitis such as AIH and are also seen in early stages of chronic biliary disorders such as PBC or PSC (Table 1). However, cholate stasis and ductopenia are specific for chronic biliary disorders and are not seen in AIH. So, in difficult cases, these findings can be investigated by the pathologist with copper stains, CK 7, and CK 19 immunostains (Table 6). In summary, close clinical-pathological cooperation is essential to reveal out the dominant disease in overlap/variant scenarios (Table 6, Figure 12).

### How Should the Variant/Overlap Syndrome Be Evaluated in Terms of Treatment Approach?

**Treatment of overlap syndrome is largely empiric. A flexible, case-based therapy selection may improve the management of difficult scenarios. An expert consult is usually needed.**

Management options for overlap syndromes include UDCA for the “cholestatic” component and immunosuppression for the “hepatitic” component. The choice and timing of treatment are determined by *the benefit/harm ratio of the treatment option*, *the predicted progression rate of the predominant disease, *and* the severity of the presentation*. Ursodeoxycholic acid given for the cholestatic component does not harm even if it is not beneficial, but the side effects of immunosuppressive drugs such as steroid, AZA, MMF, and tacrolimus given for the hepatitic component can lead to severe consequences. The initiating factor of the liver damage in the pathogenesis of AIH is autoimmune inflammation but of PBC/PSC is unclear whether it is autoimmune or caused by toxic bile or both (“chicken-egg paradox”). In AIH, the necessity of IST is clear for the “hepatitic” component, but in PBC/PSC, this decision should be made more carefully.^74-76^ If the true AIH is not given IST, the progression to cirrhosis is rapid, and severe acute cases may progress to acute liver failure, whereas the increased progression of PBC due to “hepatitic” activity is toward cirrhosis and occurs over the years in a chronic process. Moreover, inappropriate use of IST may preclude a PBC/PSC patient from newer second-line choices such as obeticholic acid or clinical new drug trial.^86^

According to the limited number of retrospective studies in PBC-AIH overlap syndrome defined by Paris criteria (ALT >5×ULN or IgG >2×ULN), combined therapy (UDCA plus IST) gives better results compared to monotherapy (UDCA), and the degree of interface hepatitis is the main indicator of “hepatitic” activity, and so combined therapy for patients with *severe* interface hepatitis and monotherapy for *moderate *interface hepatitis are recommended.^74,76,78,87^ In our opinion, since UDCA provides nearly 20% biochemical remission even in patients with severe interface hepatitis, starting UDCA first and adding IST in case of IR after 3 months is a safer approach, and considering the natural course of PBC, it does not create a disadvantage for the patient. On the other hand, if severe interface hepatitis is accompanied by hepatitic features defined by Paris criteria (ALT > 5×ULN or IgG > 2×ULN), hyperbilirubinemia, prolongation of PT/INR, or bridging necrosis, this is in favor of severe hepatitic component and a steroid trial should be started immediately.

Similar to PBC/AIH overlap, the accumulated data support the use of UDCA in combination with IST in most patients with PSC/AIH overlap syndrome despite the lack of adequate studies. In patients with severe interface hepatitis, use of immunosuppressants is mandatory. In other cases (moderate interface hepatitis), start with UDCA monotherapy and add IST only in case of inadequate biochemical response after 3 months of UDCA.^74-76^ While immunosuppression may alleviate parenchymal inflammation, this does not favorably influence bile duct injury or fibrosis. So, unlike classical AIH, inflammatory or secondary “AIH-like” flares in the longitudinal course of PSC should not deserve indefinite immunosuppression, applying of AIH treatment targets, or second-/third-line AIH treatments. Clinical evaluation and cholangiographic follow-up are better tools for de-escalation of immunosuppression over time^82^ (Illustrative Case 14).

## Non-Alcoholic Fatty Liver Disease and Autoimmune Hepatitis

The prevalence of NAFLD in the world is approximately 25% and it has become one of the leading causes of chronic liver disease.^88^ Non-alcoholic fatty liver disease spectrum consists of 95% simple steatosis and 5% NASH. Non-alcoholic fatty liver disease may be associated with other liver disorders including AIH.^89^ As expected, the prevalence of NAFLD in patients with AIH is around 25%.^90^ On the other hand, the prevalence of AIH in patients with NAFLD has not been adequately studied. When NAFLD patients were applied by scoring systems with pre-biopsy findings, a significant rate of probable and definitive AIH results were obtained, but the prevalence of AIH after biopsy decreased up to around 0.5%.^91,92^ Although the occurrence of NAFLD and AIH in the same patient is very rare and somewhat debatable, the problems in clinical practice while analyzing a patient with NAFLD and AIH features are true and not rare.

### What Are the Possible Clinical Scenarios Between Non-Alcoholic Fatty Liver Disease and Autoimmune Hepatitis?

From the view of a practicing physician, there are 3 main scenarios:

1. **Non-alcoholic fatty liver disease plus positive autoantibodies (most common scenario):** Nonspecific autoantibodies such as ANA, SMAs are seen as an epiphenomenon in a significant proportion of NAFLD patients. In this group, AIH scoring systems should not be used without liver biopsy to avoid AIH overdiagnosis in NAFLD patients (*potential risk of long-term immunosuppressive therapy*). On the other hand, given the vast number of NAFLD, non-invasive markers and Fibroscan are recommended for initial patient management without routine biopsy, and this approach may lead to underdiagnosis of AIH (*potential risk of late diagnosis of AIH at cirrhotic stage or risk of severe AIH flares*). Therefore, the presence of high IgG, fatigue, polyarthralgia, and history of other autoimmune diseases such as autoimmune thyroiditis should support a decision of liver biopsy for AIH evaluation.^31^
2. **Autoimmune hepatitis plus simple** **steatosis** **at initial presentation or later development as simple** **steatosis** **or non-alcoholic steatohepatitis (common scenario):** Concomitant steatosis is common in newly diagnosed AIH patients, especially if they have metabolic risk factors. Moreover, simple steatosis or NASH may develop by side effects of steroids during AIH treatment. It may be a reason for IR, especially if the liver test pattern is NAFLD compatible, usually normal ALP, high GGT, and ALT higher than AST.^90^
3. ****Autoimmune hepatitis** plus non-alcoholic steatohepatitis at initial presentation (rare scenario):** This is regarded as “true” AIH/NAFLD variant and carries the risk of severe liver disease. Yet, there is no consensus on its diagnostic and treatment aspects. The stronger clinicopathological criteria for both AIH and NAFLD should be present together for diagnosing a true AIH/NAFLD variant. These may include selective IgG increase, the presence of SLA, severe interface hepatitis with abundant plasma cells for AIH, and definite NASH findings on histology, IgA increase, and prominent metabolic comorbidities for NASH. On histology, the presence of hepatocyte ballooning, Mallory–Denk bodies, neutrophilic inflammation, and pericentral fibrosis, in a case of steatosis, favor definite NASH.^93^ However, inflammatory phenotypes of NASH and cirrhotic NASH may include periportal interface activity, usually lymphocytic but even with some plasma cells.^42^ Therefore, interface activity on its own does not guarantee AIH diagnosis. For example, in an asymptomatic case with mild elevation in liver enzymes, ANA positivity, IgA elevation with normal IgG, and NASH-dominant histology but also including lymphocytic interface hepatitis with occasional plasma cells favors NASH and should not be labeled as AIH/NASH overlap. Similarly, in a case with marked elevation in liver enzymes, ANA plus SLA positivity, IgG elevation, and AIH-dominant histology not only including interface hepatitis with abundant plasma cells but also including prominent steatosis without hepatocyte ballooning favors AIH with simple steatosis and should not be labeled as AIH/NASH or NASH alone. In conclusion, the diagnosis of true AIH/NASH should only be made after a careful clinicopathological analysis.

In treatment of the true AIH/NASH variant, the classical AIH scheme may need some modifications such as induction with lower doses of steroids, a short-term or more rapid tapering schedule of steroids, or the use of budesonide instead of prednisolone.^31^ Appropriate measures should also be planned for the treatment of metabolic syndrome.^94^

## Drug-Induced Liver Injury and Autoimmune Hepatitis

Drug-induced liver injury may present as every kind of liver disease phenotypes.^95^ While biochemical patterns of DILI can be hepatocellular, cholestatic or mixed, DILI with autoimmune features occurs mainly as acute or chronic hepatocellular pattern.^1^ Latency periods in these cases vary from weeks to years, irrespective of acute or chronic presentations.^96^ Moreover, a drug considered typical for immune DILI can also cause many different patterns of non-immune DILI. Therefore, many diagnostic and treatment challenges exist regarding DILI and AIH relationship. For a timely and effective management in such situations, in addition to general DILI approach including clinical and laboratory evaluation and applying of causality scores such as RUCAM, it is strongly advised reviewing the LiverTox and DILIN websites and updated literature.^4^

### What is the Context of the Drug-Induced Liver Injury and Autoimmune Hepatitis Relationship?

**Figure 13.**
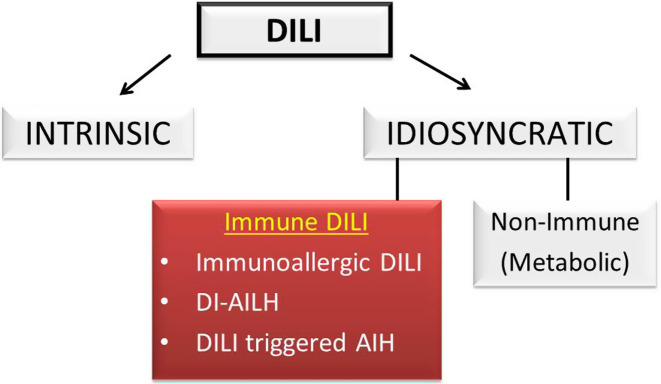
The immune-mediated drug-induced liver injury phenotypes that can resemble idiopathic autoimmune hepatitis.

Drug-induced liver injury is grouped into intrinsic and idiosyncratic forms, while idiosyncratic DILI is further subdivided into immune and non-immune forms (Figure 13).

### What Are the Main Possible Scenarios for Immune-Mediated DILI and Autoimmune Hepatitis?

Immune-mediated DILI and idiopathic AIH have overlapping diagnostic features. Immune-mediated DILI includes at least 3 distinct clinical phenotypes, immuno-allergic DILI (IA-DILI), drug-induced-autoimmune like hepatitis (DI-AILH), and true “idiopathic” chronic AIH unmasked or triggered by drugs (Table 7)^95-97^:

**Immunoallergic Drug-Induced Liver Injury** It is a drug-induced allergic hepatitis mainly caused by antibiotics, anti-retroviral agents, and anticonvulsants.^95^ Immunoallergic drug-induced liver Injury presents as a systemic reaction with hypersensitivity features such as fever, arthralgia, and skin rash, where the liver may be one of the multiple organs affected including the skin, lungs, and kidneys. Associated autoimmune antibodies can be confusing, but it is easily differentiated from DI-AILH by typical clinical and laboratory findings. Typical drug history, such as amoxicillin-clavulanate, short latent period, biochemistry findings in a cholestatic-mixed pattern, acute cholestatic hepatitis characterized by mixed inflammation and cholestasis if liver biopsy was performed, and rapid recovery when the drug is discontinued are the characteristic features. Steroid use is only indicated if there are signs of severe hypersensitivity.^95,97^
**2. Drug-Induced-Autoimmune-Like Hepatitis** Drug-induced-autoimmune-like hepatitis shares many clinical and histological features with idiopathic AIH described in previous sections, while its main difference is the absence of relapse after drug discontinuation and/or steroid treatment (Table 7). Drug-induced-autoimmune-like hepatitis typically appears with a longer latency period from initial exposure, ranging from a few weeks to months or even years for the majority of nitrofurantoin and minocycline cases, challenging of causality in relation to clinical presentation.^98,99^ Drug-induced-autoimmune-like hepatitis may present in all the clinical scenarios previously described for idiopathic AIH, but the acute presentation is relatively common. A liver biopsy is performed if the diagnosis is uncertain, if the laboratory results show severe damage, or if the steroid therapy is being planned.^4^ Although there are some histological clues in the differential diagnosis of DI-AILH vs idiopathic AIH, there is no consensus, the main criterion is the absence of relapse in DI-AILH, unlike the relapsing course of AIH^49,100,101^ (Illustrative Case 15).

The most common classical causes of DI-AILH include nitrofurantoin, minocycline, methyldopa, hydralazine, and infliximab.^102^ Drug-induced liver injury related to immune checkpoint inhibitors is not classified in DI-AILH, as liver injury of these drugs is very rarely associated with autoimmune features and classical AIH histology.^4^ Recently, Björnsson et al^103^ proposed a set of criteria for DI-AILH and performed an extensive analysis of published presumed DI-AILH reports. The suggested system is based on AIH-simplified criteria with additional items including incomplete recovery after cessation of the causative drug, need for corticosteroids to improve the liver injury, and the absence of relapse after stopping corticosteroids. To the results of the proposed criteria, interferons, imatinib, adalimumab, and methylprednisolone (not other steroids) were the other best-documented agents leading to probable DI-AILH, in addition to classical causes of DI-AILH mentioned previously. Among a substantial number of reported statin cases, only 4 of them were found as probable DI-AILH, while the majority were interpreted as a classical AIH triggered by statins, based on the relapsing course. Similarly, only 3 diclofenac-induced DI-ALH were found in old reports.

### How Is the Management Approach for Drug-Induced-Autoimmune-Like Hepatitis?

Offending drug must be discontinued. *N*-acetylcysteine and UDCA can be used for supportive therapy. There is no consensus on the necessity, timing, and dose of steroid use in DI-AILH. The severity of the disease and patient characteristics should be taken into account. In mild disease, offending drug cessation is enough for most of the patients. The main steroid indications are as follows:

1. According to Hy’s law, i.e., if serum aminotransferase levels >3-fold ULN and serum bilirubin level >2-fold ULN in hepatocellular type injury,^4,95^
2. if there is any evidence of hepatic failure like INR prolongation or deepening of jaundice as precursors of hepatic encephalopathy,
3. if clinical and laboratory findings fail to improve or worsen after 1-2 weeks of discontinuation of the offending drug, and
4. in the situation where liver histology cannot establish drug etiology with certainty.

The recommended prednisolone dose is 20-60 mg/day (oral, i.v. or even pulse with higher doses) with tapering in 3-6 months. Once remission has been achieved, withdrawal of immunosuppression and close monitoring is advised.^1,97,104,105^

### 3. True “Idiopathic” Autoimmune Hepatitis Unmasked or Triggered by Drugs

Undiagnosed AIH patients with silent course or diagnosed AIH patients in remission with low-dose therapy may experience DILI due to a new drug.^106^ This picture should be differentiated from an intrinsic flare of AIH. A detailed drug history of the last 6-12 months may reveal DILI diagnosis, while increase in serum levels of IgG in addition to serum transaminases favors AIH flare. The former scenario can usually be managed by stopping the offending drug, while that of latter needs augmentation of immunosuppression.

Finally, DILI can trigger the development of chronic AIH, as in the rare case of statins.^103^

### Pregnancy Issues in Autoimmune Hepatitis

Poorly controlled AIH is associated with amenorrhea and reduced fertility, which is restored by immunosuppression and disease control.^107^ The live birth rate is 73% in mothers with AIH, while the fetal loss and stillbirth rate of 27% is similar to that for women with other chronic diseases.^4^ Prematurity risk is increased primarily due to a flare in AIH, but there are no specific birth defects related to AIH. Similarly, the increased risk of maternal complications is mainly related to AIH flare, and it is most commonly seen in the postpartum period.

### What Are the Basic Points in Pre-Conception Counseling?

Pre-conception counseling should include a detailed information about the possible effects of AIH and drugs on the fetus and mother.^56^ The evaluation of liver disease severity and evidence of portal hypertension should be performed and the patients should be informed about the safety of usual immunosuppressive drugs used for AIH in pregnancy. Endoscopy for variceal surveillance is performed and eradication of varices is applied in advanced liver disease preferentially before conception. Propranolol is safe during pregnancy, if it is needed. Immunosuppression should be optimized, and a stable biochemical response is aimed for at least 1 year before conception. Mycophenolate mofetil is contraindicated during pregnancy due to its teratogenic risk, with 12 weeks wash-out period before conception, while an alternative drug such as tacrolimus may be preferable.^56^

### Is It Necessary to Change Immunosuppressive Drugs or Their Doses During Pregnancy? What Are the Other Important Considerations During Pregnancy?

Changing IST during pregnancy is not appropriate because of the increased risk of flare, it is continued with the same doses with standard drugs of remission (i.e., prednisolone or budesonide and AZA).^56,108^ She is monitored for loss of biochemical response which needs increase in steroid dose. Monitoring should also include gestational diabetes and pregnancy-related hypertensive disorders such as preeclampsia which may need prophylactic aspirin therapy. Re-endoscopy and MRI scan is advised to evaluate the status of varices in selected cirrhotic cases. Finally, the choice of delivery method is determined by patient preference and medical indications.

### What Are the Considerations for Postpartum Period?

This period has the highest risk of loss of biochemical response that can be effectively managed by increasing the dose of steroids. Prednisolone and AZA can safely be continued during the breastfeeding period, nursing 4 hours after drug usage. Contraception counseling is also advised.^56^

## Figures and Tables

**Table 1.** The Definitions and Pitfalls of Main Elements in Autoimmune Hepatitis Histology

	Definition	Pitfalls
**Interface hepatitis**	Spreading of inflammatory cells across the portal area into the lobule and accompanying hepatocyte apoptosis	Characteristic but not specific for AIH (i.e., marker of hepatitic injury and activity)
**Inflammatory cells**	Mainly lymphocytes, rich in plasma cells	Absence of plasma cells (only lymphocytes or even plenty of eosinophiles)
**Rosette**	Circular oriented regenerating hepatocytes around a pseudolumen	Not specific for AIH (i.e., marker of severe hepatocyte injury and rapid, extensive regeneration)
**Emperipolesis**	Penetration of lymphocyte into hepatocyte	Not specific for AIH (marker of severity)
**Ductular reaction**	Development of bile ductules via progenitor cell regenerative response	Not specific for biliary disorder (may be seen as a regenerative response in severe hepatitis or massive necrosis)
**Centrizonal necrosis**	Necroinflammation around central vein (zone 3)	Not specific for AIH (can be a marker of early/acute or severe AIH) Exclude drugs, ischemia, virus
**Lymphocytic cholangitis**	Inflammatory nondestructive bile duct injury in portal area	Not specific for biliary disorder (may be seen as an accompanying injury in AIH)
**Histological cholestasis**	Presence of bile in hepatocytes and canaliculae beginning from perivenular areas	Not specific for biliary disorder (may be seen in severe AIH and DILI)

AIH, autoimmune hepatitis; DILI, drug-induced liver injury.

**Table 2.** Scoring Systems for Autoimmune Hepatitis

Revised Diagnostic Criteria (Definite >15, Probable 10-15)	Simplified Diagnostic Criteria (Definite ≥7, Probable 6)
**C**l**inical and laboratory features** Female sex (+2) ALP/AST or ALT <1.5 (+2), 1.5-3 (0), and >3 (−2) IgG >2-fold (+3), 1-1.5 fold (+1), and < 1-fold (0) ANA, SMA, and anti-LKM-1 titers: >1 : 80 (+3), 1 : 80 (+2), 1 : 40 (+1), and <1 : 40 (0) AMA positivity: positive (−4) or negative (0) Viral markers: positive (−3) or negative (+3) Use of hepatotoxic drugs: yes (−4) or no (+1) Alcohol use: < 25 g/day (+2) or > 60 g/day daily (−2) HLADR3 or HLADR4 : positive (+1) or negative (0) Immune diseases: present (+2) or absent (0) **Histological features** Interface hepatitis (+3) Plasma cells (+1) Rosettes (+1) Absence of above 3 features (−5) Biliary changes (−3) Other features (−3) **Treatment response**: complete (+2) or relapse (+3)	**Presence of autoantibodies** ANA or SMA/anti-F-actin positive^a^ (+1) ANA or SMA/anti-F-actin strongly positive^b^ (+2) Anti-LKM-1 antibody titers of ≥1 : 40 (+2) Anti-soluble liver antigen (anti-SLA) antibody positive (+2) **Immunoglobulin level** IgG level greater than the ULN (+1) IgG level greater than >1.1 fold the ULN (+2) **Histological features** Compatible with AIH (+1) Typical of AIH (+2) **Viral hepatitis:** absent (+2) or present (0)

^a^Indirect immunofluorescence: ≥1 : 40 when assessed on tissue sections; ≥1 : 80 or 1 : 160 for ANA when assessed on HEp-2 cells, depending on local standards. ELISA with locally established cutoffs.

^b^Indirect immunofluorescence: ≥1 : 80 when assessed on tissue sections; ≥1 : 160 or 1 : 320 for ANA when assessed on HEp-2 cells. ELISA with cutoffs established locally.

(Addition of points achieve maximum 2 points for autoantibodies).

AIH, autoimmune hepatitis; ALP, alkaline phosphatase; ALT, alanine aminotransferase; AMA, anti-mitochondrial antigen; ANA, anti-nuclear autoantibodies; AST, aspartate aminotransferase; HLADR, human leukocyte antigen-DR isotype; IgG, immunoglobulin; anti-LKM, anti-liver kidney microsomal type-3; SMA, smooth muscle autoantibodies; ULN, upper limit of normal.

**Table:** 

Illustrative Case 1
A 50-year-old female presented with acute icteric hepatitis. ALT 1400, AST 1240, ALP 320, GGT 110, total bilirubin 6.5, INR 1.4. Laboratory findings were as follows; AMA negative, ANA: 1:320, IgG: 35, IgM: 8, other serologies and viral markers (negative). Diffuse portal and lobular inflammation rich in plasma cells, perivenuler cholestasis, and lymphocytic bile duct injury in some portal areas were detected in liver biopsy.
**Comment:** She had acute icteric hepatitis due to AIH. Some cholestatic features (mild increase in ALP and GGT, lymphocytic cholangitis) were due to collateral injury. Milder bile duct damage is often observed in severe portal inflammation associated with AIH. In the revised scoring system, the presence of biliary changes causes point reduction. However, these “biliary changes” targeting point reductions are restricted in bile duct changes that are typical of PBC or PSC, such as granulomatous cholangitis and severe concentric periductal fibrosis, with ductopenia and/or a substantial periportal ductular reaction with copper or copper‐binding protein accumulation. So, in the absence of AMA, PBC-specific ANA’s, and granulomatous cholangitis or ductopenia, lymphocytic cholangitis alone should not deserve PBC diagnosis. Moreover, histological cholestasis is not observed in earlier stages of PBC, but can be seen in acute or severe cases of AIH due to diffuse inflammation! In this case, remission was achieved with oral prednisolone 40 mg/d followed by 50 mg AZA at 2nd week gradually increased to 150 mg and in the 3rd year, her treatment continues with only 100 mg AZA.

**Table:** 

Illustrative Case 2
A 60-year-old female diagnosed with DM and HT had been referred to us due to elevated liver enzymes. ALT 160, AST 142, ALP 160, GGT 320, total bilirubin 0.9, INR 1.0. ANA 1:80, ASMA 1:160, IgG 15, IgM 3, other serologies and viral markers negative. USG revealed diffuse hyperechogenic liver with minimal splenomegaly.
**Comment:**If someone calculates AIH score in such a case, the result would be “probable” to the Revised system (*Female +2, ALP/ALT ratio <1.5 +2, IgG <1 ULN 0, ASMA >1:80 +3, viral negative +3, no use of drugs +1, no use of alcohol +2; 13 points*) and but “not AIH” to the Simplified system (*ASMA >1:80 +2, IgG <1 ULN 0, viral negative +2; 4 points*). But, scoring systems should not be used without biopsy as a general rule. Thereafter, a liver biopsy disclosed NASH (macrovesicular steatosis, inflammation with hepatocyte ballooning, and perivenular-pericellular fibrosis). Clinical judgement and using the scoring systems in right clinical context is essential, otherwise, a middle-aged obese woman can be diagnosed with AIH by chance in an outpatient clinic.

**Table:** 

Illustrative Case 3
A 52-year-old male patient presented with acute icteric hepatitis. ALT 1820, AST 1640, ALP 344, GGT 130, total bilirubin 7, INR 1.9. ANA, ASMA, and AMA negative, IgG 30, IgM 4, other serologies and viral markers negative. USG was normal. Centrilobular necroinflammation with a few plasma cells, lobular disarray, some ductular proliferation and mild portal infiltration were detected in liver biopsy.
**Comment:**He had acute severe hepatitis of unknown etiology. On AIH score, the result was “probable AIH” to the Revised system (*Male 0, ALP/ALT ratio <1.5 +2, IgG 1.5-2 ULN +2, ANA-ASMA negative 0, viral negative +3, no use of drugs +1, no use of alcohol +2, histology 0; 10 points*) and but “not AIH” to the Simplified system (*ANA-ASMA negative, IgG 1.5-2 ULN +2, 0, viral negative +2; histology 0; 4 points*). Centrilobular necrosis is a valuable finding in acute AIH cases, but it is not included in current scoring systems. However, as the revised system is more comprehensive, it usually diagnoses acute AIH cases. Therefore, the complementary use of scoring systems is very important.

**Table 3.** A Representative Treatment Schedule for an Adult with Autoimmune Hepatitis

Time	Prednisolone (mg/day)	Azathioprine (mg/day)
**Week 1**	40	–
**Week 2**	40	–
**Week 3**	35	50
**Week 4**	30	50
**Week 5**	25	75
**Week 6**	20	75
**Week 7**	15	100
**From week 8 to 12**	10	100
**From month 3 to 12**	7.5 > 5 > 2.5	100
**At month 12**	–	100
**At month 36**	–	–

A relatively higher prednisolone dose with slow tapering schedule is selected for an AIH patient with moderate activity.

Azathioprine is added at the third week when a decrease in transaminases is observed.

In following weeks 4-7, scheduled prednisolone tapering is continued as the ALT downward trend continues, while AZA dose is gradually increased.

Repeated normalization of ALT is secured while on 10 mg prednisolone dose between weeks 8 and 12.

From month 3 to 12, further tapering below 10 mg is continued, as a reduction of 2.5 mg per 3-month intervals, and complete withdrawal of prednisolone is achieved at the end of the first year.

Finally, AZA monotherapy is tapered and withdrawn after stable remission at the end of the third year. However, it usually relapses after a while!

**Table:** 

Illustrative Case 4
A 20-year-old male patient presented with weakness, anorexia and jaundice. ALT 485, AST 528, total bilirubin 3.5, INR 1.8, MELD score 18, ANA 1:80, SLA 1:320, IgG: 3670. Heterogeneous liver parechyma and splenomegaly (150 mm) on USG, and grade 1 varices in endoscopy, were noted. On history, at the time of appendectomy in previous year, LFT’s were high but not investigated. After performing a liver biopsy, oral prednisolone 60 mg/day was started, but stage 4 encephalopathy developed on the 6th day, while the repeat tests were as follows: ALT 158, AST 78, total bilirubin 2.4, INR 1.8, MELD score 16. Steroid was discontinued, mannitol, rifaximin and lactulose were administered, and he was listed for emergency liver transplantation. Infections were ruled out by appropriate work-up. After 3 days of supportive therapy, encephalopathy was completely resolved, and active cirrhosis was reported in liver biopsy. Meanwhile, he was diagnosed with celiac, in addition to gluten-free diet, steroid and AZA treatment was initiated again. During the following months, tacrolimus was added and partial remission could be achieved at the end of the second year, without any further clinical deterioration (ALT: 61, AST: 51, T. Bil: 1.2, INR: 1.1, IgG: 1230).
**Comment:** In this case, ACLF was triggered by intrinsic AIH activation and possibly celiac induced increased intestinal permeability and inflammation (APASL AARC score 9, ACLF grade 2, estimated survival rate at week 12, 52%. EASL CLIF-OF score 8 [No ACLF, Single cerebral failure and creatinine <1.5 mg/dl], CLIF-C-AD score 53, probability of dying at 3 months 11%). Organ function, particularly, liver, kidney, brain, lung, coagulation, and circulation should be monitored frequently and carefully throughout hospitalisation. Early identification and treatment of precipitating factors of ACLF, particularly bacterial infections, are recommended. ACLF may regress, progresses or stabilizes, so, a dynamic evaluation is required.

**Table:** 

Illustrative Case 5
A 68-year-old male patient was treated with prednisolone followed by AZA for five years with the diagnosis of AIH, but he discontinued the treatment by himself while in remission, and three years later he presented again with fatigue, anorexia and dyspeptic complaints. Hemoglobin 14, WBC 13200 (80% neutrophils), Platelets 180.000, CRP 110. ALT 300, total bilirubin 4, creatinine 1.5, INR 1.7, MELD score 21. On USG heterogeneous liver parechyma with irregular borders, diffuse ascites and splenomegaly (140 mm) was noted, no varices were detected in endoscopy, Ascitic fluid analysis was compatible with spontaneous bacterial peritonitis. Two weeks after therapy with antibiotics and iv albumin, oral prednisolone 30 mg/d and diuretics were administered. In the follow-up, ascites disappeared, AZA 50 mg/d was added to therapy and he was re-compensated thereafter. He is still being followed up with AZA monotherapy.
**Comment:** In AIH, a cessation attempt should be made after stable biochemical remission has been achieved, and preferably if the inflammation on the liver biopsy has disappeared (*Liver biopsy is not a must but optional*). Even if the cessation rules are followed, most patients relapse, usually in the first year, sometimes years later, as in this patient, so, follow-up is essential. Acute exacerbation in this patient occurred as ACLF possibly triggered by infection (APASL AARC score 6, ACLF grade 1, estimated survival rate at week 12, % 79%. EASL CLIF-OF score 6 (No ACLF, no organ failure), CLIF-C-AD score 59, probability of dying at 3 months, 19%). In ACLF-like scenarios, probable triggering factor and supportive treatment of organ failure(s) should be performed before starting immunosuppressive therapy.

**Table 4.** The Spectrum of Insufficient Response During the Course of Autoimmune Hepatitis Management

	Cause	Feature	Action
**(1) Disease-related causes **(“true” IR due to ongoing inflammation of AIH itself)	Persistent AIH activity	↑ ALT and IgG (common scenario) ↑ ALT or IgG(rare scenario)	Adjust IS therapy to current guidelines
**(2) Treatment-related causes **(IR due to insufficient immunosuppression)	Drug inadherence Drug intolerance Mistakes in treatment (i.e., inadequate dosing adjustment according to response, rapid tapering of steroid, wrong drug selection for the particular case, etc.)	↑ ALT and IgG (common scenario) ↑ ALT or IgG (rare scenario)	Secure adherence Adjust IS therapy to current guidelines
**(3) Diagnosis-related causes **(IR due to non-AIH diagnosis)	Incorrect AIH diagnosis (NASH, PBC, PSC, Wilson, etc.) Added diagnoses (NASH, DILI, viral, celiac, etc.) Variant diagnoses (PBC or PSC with AIH features) Common specific diagnoses (infections, heart failure, biliary stones, malignancies, etc.)	Compatible labs, imaging, and/or histology	Detailed work-up Specific therapy

AIH, autoimmune hepatitis; ALT, alanine aminotransferase; DILI, drug-induced liver injury; IgG, immunoglobulins; IR, insufficient response; IS, immunosuppressive; NASH, non-alcoholic steatohepatitis; PBC, primary biliary cholangitis; PSC, primary sclerosing cholangitis.

**Table 5.** Side Effects of Commonly Used Drugs in Autoimmune Hepatitis

Drug	Side Effects	Management
**Steroids**	Cosmetic: Cushingoid features Systemic: Diabetes, hypertension, fatty liver, osteoporosis, cataract, glaucoma, infections, psychosis, and depression	Taper to the lowest steroid dose needed for remission and attempt withdrawal after remission Lifestyle interventions and medical therapy for metabolic syndrome and diabetes Bone density monitoring Vitamin D and calcium administration Eye examinations for cataract and glaucoma Appropriate counselling and treatment for psychiatric issues
**AZA**	GIS: Nausea, vomiting, pancreatitis Liver: Transaminase elevation, cholestatic hepatitis, and nodular regenerative hyperplasia Hematologic: Bone marrow suppression Neoplastic: Nonmelanoma skin cancers	Check TPMT status for dose selection Monitor CBC and LFT Reduce dose if mild nausea or mild cytopenia or mild transaminase elevation occurs Discontinue in GI intolerance or severe cytopenia or marked transaminase elevation, cholestatic hepatitis, and nodular regenerative hyperplasia Avoid direct sunlight and have yearly dermatologic control
**MMF**	GIS upset, mild bone marrow suppression Rare: Severe neutropenia, pancreatitis, headache, alopecia Teratogenicity	Reduce dose if mild leukopenia or mild GI symptoms Discontinue in other scenarios Appropriate counseling and management
**Tacrolimus**	GIS upset, neurotoxicity Less common: Diabetes mellitus, nephrotoxicity, diarrhea, pruritus, and alopecia	Appropriate monitoring and management

AZA, azathioprine; CBC, complete blood count; GI, gastrointestinal; GIS, gastrointestinal system; LFT, liver function tests; MMF, mycophenolate mofetil; TPMT, thiopurine methyl transferase.

**Table:** 

Illustrative Case 9
A 45-year-old female patient presented with fatigue and jaundice. ALT 372, AST 437, total bilirubin 4.6, INR 1.4, MELD score 17, ANA 1:160, Anti-LKM 1:320, IgG: 24.5. US imaging was normal. On history, at the time of appendectomy in previous year, LFT’s were high but not investigated. After liver biopsy was performed, oral prednisolone 40 mg/day was initiated, with repeat tests performed in the second week as follows: ALT 98, AST 78, total bilirubin 1.8, INR 1.1. Then, AZA 100 mg/day was added. The patient presented with hip joint pain in the first month of treatment: ALT 28, AST 37, total bilirubin 0.8, INR 1.0. After orthopedic evaluation, MRI of the hip joint was performed, and she was diagnosed with grade 1 bilateral steroid-induced avascular femoral necrosis (AVN). She was treated by stem cell injection. The prednisolone treatment was rapidly reduced, and no increase in liver function tests was observed in the follow-up.
**Comment:** The most common drug-related cause of avascular necrosis is steroids. Although it is usually seen in young and active male patients, every patient receiving steroid therapy should be followed up for AVN.

**Table:** 

Illustrative Case 10
A 34-year-old female patient presented with fatigue. ALT 172, AST 187, total bilirubin 1.0, INR 0.9, ANA 1:80, SMA 1:160, IgG 21. US imaging was normal. On history, serum aminotransferases were found to be high for 2 years. Liver biopsy was compatible with AIH and she was started on 40 mg prednisolone and subsequent AZA treatment. In the third week of AZA treatment, she was admitted to the hospital with fever and diagnosed with AZA-related neutropenia and secondary infection. AST 98, ALT 110, total bilirubin 1.0, INR 0.9, WBC 2100X103, PNL 300, Hb 14.5 gr/dl, Plt 190x106. AZA was discontinued and MMF 1000 mg/d was started in the post-infection period. Complete biochemical remission was achieved in the 6th month of treatment.
**Comment:** When taken at a reasonable dose, AZA rarely causes bone marrow suppression, but it can have serious consequences. Regular monitoring with CBC is warranted.

**Table:** 

Illustrative Case 11
A 32-year-old female presented with fatigue and pruritus for 6 months. ALT 133, AST 147, ALP 620, GGT 702, total bilirubin 0.8, INR 1.1. AMA 1:320, ANA 1:160, IgG 19, IgM 7, other serologies and viral markers were negative. Portal lymphoplasmocytic infiltration, moderate interface hepatitis and granulomatous cholangitis were detected in liver biopsy.
**Comment:**In this patient, hepatitic (ALT, ANA, IgG, interface hepatitis) and cholestatic features (ALP, AMA, IgM, granulomatous cholangitis) are together, but it is clear that the dominant disease is PBC. According to the consensus report, the correct label should be PBC with some hepatitic features (serological and histological overlap), and she was put on UDCA treatment.
According to the consensus report, the use of AIH scoring systems, and even the Paris criteria, is not recommended in the diagnosis of “overlap syndrome” but the latter is recommended in EASL and other guidelines. If used, she would get probable diagnosis of AIH (revised system 10 points, simplified system 6 point), and the diagnosis of “PBC plus AIH overlap syndrome” to the Paris criteria.

**Table:** 

Illustrative Case 12
A 45-years-old female with fatigue and dry eyes for two years. ALT 255, AST 160, ALP 455, GGT 322, total bilirubin 0.8, INR 1. AMA negative, AMA-M2 negative, ANA 1:80, IgG 21, IgM 6.3, other serologies and viral markers were negative.
**Comment:**She had some cholestatic and hepatitic features together. According to the revised and simplified scoring systems AIH score would be “probable”. But, *scoring systems should not be used without biopsy as a general rule.* Thereafter, a percutaneous liver biopsy disclosed destructive granulomatous cholangitis and moderate lymphocytic interface hepatitis. The biopsy findings are not found to be consistent with the diagnosis of AIH. Other possible misdiagnoses would be “autoimmune cholangitis” because of AMA negativity, or “PBC-AIH overlap syndrome” because of combined histological (destructive granulomatous cholangitis and moderate interface hepatitis) and laboratory findings (ALT>5 times, ALP>2 times) consistent with Paris criteria. Further evaluation showed Gp210 positivity, review of histology was interpreted as PBC stage 2 and UDCA response was excellent. The diagnosis is currently “AMA negative PBC with some hepatitic features”.

**Table:** 

Illustrative Case 13
First admission: A 38-year-old female admitted with nausea and vomiting. ALT 800, AST 1000, ALP 150, T. Bil 3, PTZ 14 sn. Laparoscopic cholecystectomy was performed for cholelithiasis detected on abdominal USG. Second admission: At post-op 2nd week nausea and vomiting continued, and jaundice developed. Liver tests were found similar to those in the first hospitalization. USG normal, ANA 1:80, AMA 1: 320, IgG 30, IgM 3.1. In liver biopsy, portal inflammation and ductular proliferation were interpreted as PBC and UDCA was started. Third admission: She was re-hospitalized at the 8th week due to the development of encephalopathy and hypoglycemia. ALT 300, AST 970, ALP 130, T. Bil 18, PTZ 25 sec. On USG, heterogeneous liver and ascites was seen; ANA 1:80, AMA 1:160, ASMA positive. Liver transplantation was performed. Panlobular inflammation and bridging necrosis were reported in the first liver biopsy, while massive collapse and regeneration nodules, without cirrhosis were observed in the explant liver.
**Comment:** First admission: When the patient presented with acute icteric hepatitis, the operation was performed since cholelithiasis was detected on USG, despite the absence of symptoms such as biliary colic or signs of acute cholecystitis such as thickening of the gallbladder wall and leukocytosis. Second admission: Jaundice is not expected in early stage non-cirrhotic PBC, and it does not present as acute icteric hepatitis. In addition, ductular proliferation in liver biopsy is a nonspecific sign of regeneration, and together with false positive AMA, it was mistakenly interpreted in favor of PBC.
Third admission: With the addition of coagulopathy and encephalopathy, ALF developed in the subacute process in the patient and liver transplantation was performed. When the first biopsy and explant were examined, it was observed that severe acute hepatitis that started with widespread necroses progressed in the subacute phase with collapse and regenerative nodules. If acute severe hepatitis due to AIH was diagnosed earlier and steroid therapy was initiated, remission could probably be achieved without transplantation. False positivity of serum AMA and transglutaminases have been frequently reported in acute liver failure.

**Table 6.** The Main Differentials in Overlap/Variant Scenarios^*^

	AIH	PBC	PSC
**Clinical**	Jaundice may be seen in early stages ALF may complicate in severe cases	No jaundice in early stages No ALF	Fluctuating jaundice Acute or recurrent cholangitis IBD No ALF
**Serology**	SLA	PBC-specific ANA’s (sp100, gp210) High titer AMA-M2	pANCA
**Histology**	Severe interface hepatitis with lobular inflammation confluent necrosis (centrilobular to massive)	Florid ductal lesion Granulomatous cholangitis Ductopenia Cholate stasis	Fibrosing cholangitis (periductal concentric fibrosis, fibrotic nodule in portal area) ductopenia Cholate stasis
	Histological cholestasis may present in early stages (in acute severe forms)	Histological cholestasis absent in early stages	Histological cholestasis may present in early stages (as fluctuating manner)
	Chronic biliary features are absent	Chronic biliary features are present (copper, CK7, CK19 positivity)	Chronic biliary features are present (copper, CK7, CK19 positivity)
**Radiology**	–	–	Strictures on MRCP, ERCP

^*^The features mentioned in clinical, histology, and radiology apply for only non-cirrhotic stages.

ALF, acute liver failure; ANA, anti-nuclear autoantibodies; Ck7, cytokeratin-7; ERCP, endoscopic retrograde cholangiopancreatography; IBD, inflammatory bowel disease; MRCP, magnetic resonance cholangiopancreatography.

**Table:** 

Illustrative Case 14
A 35-years-old male with fatique and anorexia for three months. ALT 165, AST 140, ALP 210, GGT 120, total bilirubin 1.2, INR 0.9. ANA 1:40, ASMA 1:40, IgG 23, IgM 2.4, other serologies and viral markers negative. USG was normal. Portal lymphoplasmocytic infiltration and moderate interface hepatitis were noted in liver biopsy. With the diagnosis of AIH, oral prednisolone 60 mg/d followed by AZA 100 mg/d was started. While ALT, AST and IgG returned to normal in three months, ALP and GGT only slightly decreased. Meanwhile, MRCP was requested when the patient had two attacks of right upper quadrant pain, fever and chills. Findings were compatible with large-duct PSC, and UDCA 15 mg/kg and cipro 2x500 mg po were started. Although cholestasis enzymes were partially reduced, biliary colic attacks continued and palliation was performed by dilatation with ERCP. Though strictures have progressed on cholangiography, he is still being followed up with intermittent dilatation therapy after five years of diagnosis. Immunosuppressive drugs were withdrawn.
**Comment:**In this case, the initial laboratory tests and biopsy findings were compatible with AIH and regressed with immunosuppressive therapy, but, the increase in cholestasis enzymes and the recurrent cholangitis suggested the underlying PSC. As AIH evolves to PSC clinically, typical histological features of AIH such as portal inflammation and interface hepatitis tend to subside, likely reflecting successful therapeutic suppression of inflammation, and features of chronic biliary disease such as biliary interface activity and ductopenia become more prominent. Classical ‘onion-skin’ fibrosis mainly involve medium sized ducts and seen infrequently in needle biopsy specimens. A longitudinal approach to care must be adopted when managing overlap syndrome to clarify diagnosis and judge for treatment. This is particularly relevant in patients with PSC and overlapping AIH features in whom long-term immunosuppression not supported by evidence of benefit.

**Table 7.** The Features of Main Immune Drug-Induced Liver Injury Phenotypes versus True Autoimmune Hepatitis

	Immunoallergic DILI	DI-AILH	True AIH
**Typical drug**	Amoxicillin/clavulanate etc.	Nitrofurantoin etc.	Idiopathic
**Latency**	Short (days–weeks)	Variable (weeks–months–years)	Long (years)
**Biochemistry**	Cholestatic, mixed > hepatitic (lymphocytosis, eosinophilia)	Acute or chronic hepatitis	Chronic or acute hepatitis
**Serology**	ANA, ASMA may be positive	ANA, ASMA, IgG	ANA, ASMA, IgG
**Histology**	Mixed inflammation	Severe inflammationCirrhosis rare	Severe inflammationCirrhosis not rare
**Management**	Stop drug, steroid (rare)	Stop drug, steroid	Steroid/AZA
**Course**	No relapse	No relapse	Relapse

**Table:** 

Illustrative Case 15
A 48-year-old male patient presented with nause and abdominal pain. ALT 472, AST 387, total bilirubin 11.3, INR 1.4, ANA 1:160, IgG: 1650. He has a history of drinking herbal tea and NSAIDs for 3 weeks. Viral hepatitis serology was negative. Following the liver biopsy, prednisolone 40 mg/d treatment was started. In tests performed on the 7th day of treatment: AST 47, ALT 50, T.bilirubin 1.0, INR:0.9 Liver biopsy: Mild-moderate interface hepatitis, neutrophils and eosinophils in the portal tracts and intracellular cholestasis. Provisional diagnosis was immune-mediated DILI. No relapse was observed in the long-term follow-up of the patient whose steroid treatment was rapidly discontinued.
**Comment:** DI-AILH is a difficult diagnosis to differentiate from AIH clinically and serologically. Differentiating between DI-AILH and idiopathic AIH, particularly when AIH is seronegative or when serum IgG levels are normal, is a common clinical problem. Although there are some histological clues in the differential diagnosis of DI-AILH vs idiopathic AIH, there is no consensus, the main criterion is the absence of relapse in DI-AILH, unlike the relapsing course of AIH.

## References

[1] European Association for the Study of the Liver. EASL Clinical Practice Guidelines: autoimmune hepatitis. J Hepatol. 2015;63(4):971 1004. (10.10.1016/j.jhep.2015.06.030) 26341719

[2] Dyson JK Wong LL Bigirumurame T , et al. Inequity of care provision and outcome disparity in autoimmune hepatitis in the United Kingdom. Aliment Pharmacol Ther. 2018;48(9):951 960. (10.10.1111/apt.14968) 30226274 PMC6667893

[3] Liberal R de Boer YS Andrade RJ , et al. Expert clinical management of autoimmune hepatitis in the real world. Aliment Pharmacol Ther. 2017;45(5):723 732. (10.10.1111/apt.13907) 28004405

[4] Mack CL Adams D Assis DN , et al. Diagnosis and management of autoimmune hepatitis in adults and children: 2019 practice guidance and guidelines from the American Association for the Study of Liver Diseases. Hepatology. 2020;72(2):671 722. (10.10.1002/hep.31065) 31863477

[5] Dalekos GN Azariadis K Lygoura V Arvaniti P Gampeta S Gatselis NK . Autoimmune hepatitis in patients aged 70 years or older: disease characteristics, treatment response and outcome. Liver Int. 2021;41(7):1592 1599. (10.10.1111/liv.14900) 33896089

[6] Rahim MN Miquel R Heneghan MA . Approach to the patient with acute severe autoimmune hepatitis. JHEP Rep. 2020;2(6):100149. (10.10.1016/j.jhepr.2020.100149) 32995712 PMC7509236

[7] Wendon J Cordoba J Dhawan KA , et al. EASL Clinical Practical Guidelines on the management of acute (fulminant) liver failure. J Hepatol. 2017;66:1047 1081. 28417882 10.1016/j.jhep.2016.12.003

[8] Fujiwara K Yasui S Yokosuka O . Autoimmune acute liver failure: an emerging etiology for intractable acute liver failure. Hepatol Int. 2013;7(2):335 346. (10.10.1007/s12072-012-9402-3) 26201768

[9] Ozaslan E Efe C . Further considerations in autoimmune hepatitis. J Hepatol. 2016;64(6):1457 1458. (10.10.1016/j.jhep.2015.12.027) 26916527

[10] Zachou K Arvaniti P Azariadis K , et al. Prompt initiation of high-dose i.v. corticosteroids seem to prevent progression to liver failure in patients with original acute severe autoimmune hepatitis. Hepatol Res. 2019;49(1):96 104. (10.10.1111/hepr.13252) 30248210

[11] Dalekos GN Gatselis NK Zachou K . Acute severe autoimmune hepatitis: corticosteroids or liver transplantation? Liver Transpl. 2019;25(10):1588 1589. (10.10.1002/lt.25615) 31359585

[12] Yasui S Fujiwara K Okitsu K Yonemitsu Y Ito H Yokosuka O . Importance of computed tomography imaging features for the diagnosis of autoimmune acute liver failure. Hepatol Res. 2012;42(1):42 50. (10.10.1111/j.1872-034X.2011.00892.x) 21988323

[13] Moreau R Jalan R Gines P , et al. Acute-on-chronic liver failure is a distinct syndrome that develops in patients with acute decompensation of cirrhosis. Gastroenterology. 2013;1441:1426 1437. 10.1053/j.gastro.2013.02.042 23474284

[14] European Association for the Study of the Liver. EASL Clinical Practice Guidelines on acute-on-chronic liver failure. J Hepatol. 2023;79(2):461 491. (10.10.1016/j.jhep.2023.04.021) 37364789

[15] Sarin SK Choudhury A Sharma MK , et al. Acute-on-chronic liver failure: consensus recommendations of the Asian Pacific association for the study of the liver (APASL): an update. Hepatol Int. 2019;13(4):353 390. (10.10.1007/s12072-019-09946-3) 31172417 PMC6728300

[16] Jalan R Saliba F Pavesi M , et al. Development and validation of a prognostic score to predict mortality in patients with acute-on-chronic liver failure. J Hepatol. 2014;61(5):1038 1047. (10.10.1016/j.jhep.2014.06.012) 24950482

[17] Bajaj JS O’Leary JG Reddy KR , et al. Survival in infection-related acute on- chronic liver failure is defined by extrahepatic organ failures. Hepatology. 2014;60(1):250 256. (10.10.1002/hep.27077) 24677131 PMC4077926

[18] Bajaj JS O’Leary JG Lai JC , et al. Acute-on- chronic liver failure clinical guidelines. Am J Gastroenterol. 2022;117(2):225 252. (10.10.14309/ajg.0000000000001595) 35006099

[19] Dhaliwal HK Hoeroldt BS Dube AK , et al. Long-term prognostic significance of persisting histological activity despite biochemical remission in autoimmune hepatitis. Am J Gastroenterol. 2015;110(7):993 999. (10.10.1038/ajg.2015.139) 26010310

[20] Johnson PJ McFarlane IG . International Autoimmune Hepatitis Group Report: review of criteria for diagnosis of autoimmune hepatitis. Hepatology. 1993;18(4):998 1005. (10.10.1002/hep.1840180435) 8406375

[21] Alvarez F Berg PA Bianchi FB , et al. International Autoimmune Hepatitis Group Report: review of criteria for diagnosis of autoimmune hepatitis. J Hepatol. 1999;31(5):929 938. (10.10.1016/s0168-8278(99)80297-9) 10580593

[22] Czaja AJ Carpenter HA . Autoimmune hepatitis overlap syndromes and liver pathology. Gastroenterol Clin North Am. 2017;46(2):345 364. (10.10.1016/j.gtc.2017.01.008) 28506369

[23] Czaja AJ . Cholestatic phenotypes of autoimmune hepatitis. Clin Gastroenterol Hepatol. 2014;12(9):1430 1438. (10.10.1016/j.cgh.2013.08.039) 24013108

[24] Sun SM Wang YY Zhang Q , et al. Serum levels of immunoglobulins in an adult population and their relationship with nonalcoholic fatty liver disease. J Dig Dis. 2018;19(8):498 507. (10.10.1111/1751-2980.12646) 29989347

[25] Hartl J Miquel R Zachou K , et al. Features and outcome of AIH patients without elevation of IgG. JHEP Rep. 2020;2(3):100094. (10.10.1016/j.jhepr.2020.100094) 32280942 PMC7139106

[26] Dalekos GN Gatselis NK . Autoimmune serology testing in clinical practice: an updated roadmap for the diagnosis of autoimmune hepatitis. Eur J Intern Med. 2023;108:9 17. (10.10.1016/j.ejim.2022.11.013):() 36400668

[27] Gleeson D Heneghan MA , British Society of Gastroenterology. British Society of Gastroenterology (BSG) guidelines for management of autoimmune hepatitis. Gut. 2011;60(12):1611 1629. (10.10.1136/gut.2010.235259) 21757447

[28] Dalekos GN Koskinas J Papatheodoridis GV . Hellenic Association for the Study of the Liver Clinical Practice Guidelines: autoimmune hepatitis. Ann Gastroenterol. 2019;32(1):1 23. (10.10.20524/aog.2018.0330) 30598587 PMC6302199

[29] Terziroli Beretta-Piccoli B Mieli-Vergani G Vergani D . Serology in autoimmune hepatitis: a clinical-practice approach. Eur J Intern Med. 2018;48:35 43. (10.10.1016/j.ejim.2017.10.006) 29056396

[30] Galaski J Weiler-Normann C Schakat M , et al. Update of the simplified criteria for autoimmune hepatitis: evaluation of the methodology for immunoserological testing. J Hepatol. 2021;74(2):312 320. (10.10.1016/j.jhep.2020.07.032) 32730794

[31] Dalekos GN Gatselis NK Zachou K Koukoulis GK . NAFLD and autoimmune hepatitis: do not judge a book by its cover. Eur J Intern Med. 2020;75:1 9. (10.10.1016/j.ejim.2020.02.001) 32051092

[32] Tan EM Feltkamp TE Smolen JS , et al. Range of antinuclear ­antibodies in “healthy” individuals. Arthritis Rheum. 1997;40(9):1601 1611. (10.10.1002/art.1780400909) 9324014

[33] Czaja AJ . Performance parameters of the conventional serological markers for autoimmune hepatitis. Dig Dis Sci. 2011;56(2):545 554. (10.10.1007/s10620-010-1501-1) 21127976

[34] Granito A Muratori P Muratori L , et al. Antinuclear antibodies giving the ‘multiple nuclear dots’ or the ‘rim-like/membranous’ patterns: diagnostic accuracy for primary biliary cirrhosis. Aliment Pharmacol Ther. 2006;24(11-12):1575 1583. (10.10.1111/j.1365-2036.2006.03172.x) 17206945

[35] Bogdanos DP Komorowski L . Disease-specific autoantibodies in primary biliary cirrhosis. Clin Chim Acta. 2011;412(7-8):502 512. (10.10.1016/j.cca.2010.12.019) 21185272

[36] Wang G Tanaka A Zhao H , et al. The Asian Pacific Association for the Study of the Liver clinical practice guidance: the diagnosis and management of patients with autoimmune hepatitis. Hepatol Int. 2021;15(2):223 257. (10.10.1007/s12072-021-10170-1) 33942203 PMC8144150

[37] Batts KP . Autoimmune and chronic cholestatic disorders of the liver. In: Odze R Goldblum JR , eds. Odze and Goldblum Surgical Pathology of the GI Tract, Liver, Biliary Tract and Pancreas. 3rd ed. Amsterdam: Elsevier; 2015:1262 1284.

[38] Laschtowitz A Zachou K Lygoura V , et al. Histological activity despite normal ALT and IgG serum levels in patients with autoimmune hepatitis and cirrhosis. JHEP Rep. 2021;3(4):100321. (10.10.1016/j.jhepr.2021.100321) 34381983 PMC8333110

[39] Balitzer D Shafizadeh N Peters MG Ferrell LD Alshak N Kakar S . Autoimmune hepatitis: review of histologic features included in the simplified criteria proposed by the international autoimmune hepatitis group and proposal for new histologic criteria. Mod Pathol. 2017;30(5):773 783. (10.10.1038/modpathol.2016.267) 28106105

[40] Zachou K Azariadis K Sofia M , et al. Acute non-A, non-B, non-C hepatitis differences and similarities between hepatitis E virus infection and autoimmune hepatitis, with phylogenetic analysis of hepatitis E virus in humans and wild boars. Ann Gastroenterol. 2022;35(5):532 540. (10.10.20524/aog.2022.0731) 36061156 PMC9399571

[41] Ozaslan E Efe C Gokbulut Ozaslan N . The diagnosis of antimitochondrial antibody-negative primary biliary cholangitis. Clin Res Hepatol Gastroenterol. 2016;40(5):553 561. (10.10.1016/j.clinre.2016.06.001) 27567165

[42] Flatley S Dube AK Gleeson D . Histopathologist and clinician interface in diagnosis and management of autoimmune hepatitis. Frontline Gastroenterol. 2022;13(e1):e94 e101. (10.10.1136/flgastro-2022-102192) 35812025 PMC9234737

[43] de Boer YS van Nieuwkerk CM Witte BI Mulder CJ Bouma G Bloemena E . Assessment of the histopathological key features in autoimmune hepatitis. Histopathology. 2015;66(3):351 362. (10.10.1111/his.12558) 25257662

[44] Verdonk RC Lozano MF van den Berg AP Gouw AS . Bile ductal injury and ductular reaction are frequent phenomena with different significance in autoimmune hepatitis. Liver Int. 2016;36(9):1362 1369. (10.10.1111/liv.13083) 26849025

[45] Carpenter HA Czaja AJ . The role of histologic evaluation in the diagnosis and management of autoimmune hepatitis and its variants. Clin Liver Dis. 2002;6(3):685 705. (10.10.1016/s1089-3261(02)00022-3) 12362575

[46] Lefkowitch JH . Cholestasis. In: Ferrell LD Kakar S , eds. Liver Pathology. 1st ed. USA: Demos Medical Publishing; 2011:89 95.

[47] Alvarez F Berg PA Bianchi FB , et al. [report]. International Autoimmune Hepatitis Group Report: review of criteria for diagnosis of autoimmune hepatitis. J Hepatol. 1999;31(5):929 938. (10.10.1016/s0168-8278(99)80297-9) 10580593

[48] Hennes EM Zeniya M Czaja AJ , et al. Simplified criteria for the diagnosis of autoimmune hepatitis. Hepatology. 2008;48(1):169 176. (10.10.1002/hep.22322) 18537184

[49] Lohse AW Sebode M Bhathal PS , et al. Consensus recommendations for histological criteria of autoimmune hepatitis from the International AIH Pathology Group: results of a workshop on AIH histology hosted by the European Reference Network on Hepatological Diseases and the European Society of Pathology: results of a workshop on AIH histology hosted by the European Reference Network on Hepatological Diseases and the European Society of Pathology. Liver Int. 2022;42(5):1058 1069. (10.10.1111/liv.15217) 35230735

[50] Ozaslan E . Histologic criteria of autoimmune hepatitis: is there anything left to discuss? Liver Int. 2022;42(11):2587 2588. (10.10.1111/liv.15391) 35946048

[51] Czaja AJ Freese DK , American Association for the Study of Liver Disease. Diagnosis and treatment of autoimmune hepatitis. Hepatology. 2002;36(2):479 497. (10.10.1053/jhep.2002.34944) 12143059

[52] Manns MP Czaja AJ Gorham JD , et al. Diagnosis and management of autoimmune hepatitis. Hepatology. 2010;51(6):2193 2213. (10.10.1002/hep.23584) 20513004

[53] Czaja AJ . Features and consequences of untreated type 1 autoimmune hepatitis. Liver Int. 2009;29(6):816 823. (10.10.1111/j.1478-3231.2008.01904.x) 19018980

[54] Wang Z Sheng L Yang Y , et al. The management of autoimmune hepatitis patients with decompensated cirrhosis: real-world experience and a comprehensive review. Clin Rev Allergy Immunol. 2017;52(3):424 435. (10.10.1007/s12016-016-8583-2) 27515672

[55] Janmohamed A Hirschfield GM . Autoimmune hepatitis and complexities in management. Frontline Gastroenterol. 2019;10(1):77 87. (10.10.1136/flgastro-2018-101015) 30651962 PMC6319158

[56] Chung YY Heneghan MA . Autoimmune hepatitis in pregnancy: pearls and pitfalls. Hepatology. 2022;76(2):502 517. (10.10.1002/hep.32410) 35182079

[57] Pape S Snijders RJALM Gevers TJG , et al. Systematic review of response criteria and endpoints in autoimmune hepatitis by the International Autoimmune Hepatitis Group. J Hepatol. 2022;76(4):841 849. (10.10.1016/j.jhep.2021.12.041) 35066089

[58] Pape S Gevers TJG Belias M , et al. Predniso(lo)ne dosage and chance of remission in patients with autoimmune hepatitis. Clin Gastroenterol Hepatol. 2019;17(10):2068 2075.e2. (10.10.1016/j.cgh.2018.12.035) 30625402

[59] Dalekos GN Arvaniti P Gatselis NK , et al. First Results from a Propensity Matching Trial of mycophenolate mofetil vs. azathioprine in Treatment-Naive AIH Patients. Front Immunol. 2021;12:798602. (10.10.3389/fimmu.2021.798602) 35087524 PMC8787111

[60] Czaja AJ Menon KV Carpenter HA . Sustained remission after corticosteroid therapy for type 1 autoimmune hepatitis: a retrospective analysis. Hepatology. 2002;35(4):890 897. (10.10.1053/jhep.2002.32485) 11915036

[61] Harrison L Gleeson D . Stopping immunosuppressive treatment in autoimmune hepatitis (AIH): is it justified (and in whom and when)? Liver Int. 2019;39(4):610 620. (10.10.1111/liv.14051) 30667576

[62] Hartl J Ehlken H Weiler-Normann C , et al. Patient selection based on treatment duration and liver biochemistry increases success rates after treatment withdrawal in autoimmune hepatitis. J Hepatol. 2015;62(3):642 646. (10.10.1016/j.jhep.2014.10.018) 25457202

[63] Lohse AW Sebode M Jørgensen MH , et al. Second-line and third-line therapy for autoimmune hepatitis: a position statement from the European Reference Network on Hepatological Diseases and the International Autoimmune Hepatitis Group. J Hepatol. 2020;73(6):1496 1506. (10.10.1016/j.jhep.2020.07.023) 32707224

[64] Zachou K Weiler-Normann C Muratori L Muratori P Lohse AW Dalekos GN . Permanent immunosuppression in SLA/LP-positive autoimmune hepatitis is required although overall response and survival are similar. Liver Int. 2020;40(2):368 376. (10.10.1111/liv.14280) 31626725

[65] Rahim MN Liberal R Miquel R Heaton ND Heneghan MA . Acute severe autoimmune hepatitis: corticosteroids or liver transplantation? Liver Transpl. 2019;25(6):946 959. (10.10.1002/lt.25451) 30900368

[66] Yeoman AD Westbrook RH Zen Y , et al. Early predictors of corticosteroid treatment failure in icteric presentations of autoimmune hepatitis. Hepatology. 2011;53(3):926 934. (10.10.1002/hep.24141) 21374663

[67] De Martin E Coilly A Chazouillères O , et al. Early liver transplantation for corticosteroid non-responders with acute severe autoimmune hepatitis: the SURFASA score. J Hepatol. 2021;74(6):1325 1334. (10.10.1016/j.jhep.2020.12.033) 33503489

[68] Anand L Choudhury A Bihari C , et al. Flare of autoimmune hepatitis causing acute on chronic liver failure: diagnosis and response to corticosteroid therapy. Hepatology. 2019;70(2):587 596. (10.10.1002/hep.30205) 30113706

[69] Sharma S Agarwal S Gopi S , et al. Determinants of outcomes in autoimmune hepatitis presenting as acute on chronic liver failure without extrahepatic organ dysfunction upon treatment with steroids. J Clin Exp Hepatol. 2021;11(2):171 180. (10.10.1016/j.jceh.2020.08.007) 33746441 PMC7953011

[70] Efe C Hagström H Ytting H , et al. Efficacy and safety of mycophenolate mofetil and tacrolimus as second-line therapy for patients with autoimmune hepatitis. Clin Gastroenterol Hepatol. 2017;15(12):1950 1956.e1. (10.10.1016/j.cgh.2017.06.001) 28603052

[71] Chung Y Rahim MN Graham JJ Zen Y Heneghan MA . An update on the pharmacological management of autoimmune hepatitis. Expert Opin Pharmacother. 2021;22(11):1475 1488. (10.10.1080/14656566.2021.1895747) 33624559

[72] van den Brand FF van der Veen KS Lissenberg-Witte BI , et al. Adverse events related to low dose corticosteroids in autoimmune hepatitis. Aliment Pharmacol Ther. 2019;50(10):1120 1126. (10.10.1111/apt.15528) 31617229 PMC6899908

[73] Pape S Gevers TJG Vrolijk JM , et al. High discontinuation rate of azathioprine in autoimmune hepatitis, independent of time of treatment initiation. Liver Int. 2020;40(9):2164 2171. (10.10.1111/liv.14513) 32410363 PMC7496382

[74] Boberg KM Chapman RW Hirschfield GM , et al. Overlap syndromes: the International Autoimmune Hepatitis Group (IAIHG) position statement on a controversial issue. J Hepatol. 2011;54(2):374 385. (10.10.1016/j.jhep.2010.09.002) 21067838

[75] Czaja AJ . The overlap syndromes of autoimmune hepatitis. Dig Dis Sci. 2013;58(2):326 343. (10.10.1007/s10620-012-2367-1) 22918690

[76] Schulz L Sebode M Weidemann SA Lohse AW . Variant syndromes of primary biliary cholangitis. Best Pract Res Clin Gastroenterol. 2018;34 -35:55 61. (10.10.1016/j.bpg.2018.06.003) 30343711

[77] Imanieh M Farzaneh NA Dehghani SM Shahrebabak MG Hosseinabadi SH . Evaluation of validity and efficiency of diagnostic criteria in autoimmune hepatitis in children. Turk J Gastroenterol. 2021;32(6):526 531. (10.10.5152/tjg.2021.19305) 34405819 PMC8975465

[78] Lohse AW zum Büschenfelde KH Franz B Kanzler S Gerken G Dienes HP . Characterization of the overlap syndrome of primary biliary cirrhosis (PBC) and autoimmune hepatitis: evidence for it being a hepatitic form of PBC in genetically susceptible individuals. Hepatology. 1999;29(4):1078 1084. (10.10.1002/hep.510290409) 10094950

[79] Chazouillères O Wendum D Serfaty L Montembault S Rosmorduc O Poupon R . Primary biliary cirrhosis–autoimmune hepatitis overlap syndrome: clinical features and response to therapy. Hepatology. 1998;28(2):296 301. (10.10.1002/hep.510280203) 9695990

[80] European Association for the Study of the Liver. EASL Clinical Practice Guidelines: the diagnosis and management of patients with primary biliary cholangitis. J Hepatol. 2017;67(1):145 172. (10.10.1016/j.jhep.2017.03.022) 28427765

[81] Mieli-Vergani G Vergani D Baumann U , et al. Diagnosis and management of pediatric autoimmune liver disease: ESPGHAN hepatology committee position statement. J Pediatr Gastroenterol Nutr. 2018;66(2):345 360. (10.10.1097/MPG.0000000000001801) 29356770

[82] Ricciuto A Kamath BM Hirschfield GM Trivedi PJ . Primary sclerosing cholangitis and overlap features of autoimmune hepatitis: a coming of age or an age-ist problem? J Hepatol. 2023;79(2):567 575. (10.10.1016/j.jhep.2023.02.030) 36870613

[83] van Leeuwen DJ Sood G Ferrante D Lazenby AJ Sellers MJ . A 38-year-old African-American woman with an unusually rapid progression of “Primary Biliary Cirrhosis”: a missed opportunity! Semin Liver Dis. 2002;22(4):395 406. (10.10.1055/s-2002-35710) 12447711

[84] Portmann B Zen Y . Inflammatory disease of the bile ducts-cholangiopathies: liver biopsy challenge and clinicopathological correlation. Histopathology. 2012;60(2):236 248. (10.10.1111/j.1365-2559.2011.03853.x) 21668470

[85] Washington K . Bile duct damage and ductopenia. In: Ferrell L Kakar S , eds. Liver Pathology. 1st ed. N ew York: Demos M edical; 2011:117 130.

[86] Corrigan M Hirschfield GM . Autoimmune liver disease: evaluating overlapping and cross-over presentations-a case-based discussion. Frontline Gastroenterol. 2016;7(4):240 245. (10.10.1136/flgastro-2016-100698) 28839864 PMC5369510

[87] Ozaslan E Efe C Heurgué-Berlot A , et al. Factors associated with response to therapy and outcome of patients with primary biliary cirrhosis with features of autoimmune hepatitis. Clin Gastroenterol Hepatol. 2014;12(5):863 869. (10.10.1016/j.cgh.2013.09.021) 24076417

[88] Younossi ZM Koenig AB Abdelatif D Fazel Y Henry L Wymer M . Global epidemiology of nonalcoholic fatty liver disease—metaanalytic assessment of prevalence, incidence, and outcomes. Hepatology. 2016;64(1):73 84. (10.10.1002/hep.28431) 26707365

[89] Takahashi A Arinaga-Hino T Ohira H , et al. Non-alcoholic fatty liver disease in patients with autoimmune hepatitis. JGH Open. 2018;2(2):54 58. (10.10.1002/jgh3.12046) 30483564 PMC6207019

[90] De Luca-Johnson J Wangensteen KJ Hanson J Krawitt E Wilcox R . Natural history of patients presenting with autoimmune hepatitis and coincident nonalcoholic fatty liver disease. Dig Dis Sci. 2016;61(9):2710 2720. (10.10.1007/s10620-016-4213-3) 27262844 PMC6357773

[91] Adams LA Lindor KD Angulo P . The prevalence of autoantibodies and autoimmune hepatitis in patients with nonalcoholic fatty liver disease. Am J Gastroenterol. 2004;99(7):1316 1320. (10.10.1111/j.1572-0241.2004.30444.x) 15233671

[92] Yatsuji S Hashimoto E Kaneda H Taniai M Tokushige K Shiratori K . Diagnosing autoimmune hepatitis in nonalcoholic fatty liver disease: is the International Autoimmune Hepatitis Group ­scoring system useful? J Gastroenterol. 2005;40(12):1130 1138. (10.10.1007/s00535-005-1711-z) 16378177

[93] Brunt EM Kleiner DE Carpenter DH , et al. NAFLD: Reporting ­Histologic Findings in Clinical Practice. Hepatology. 2021;73(5):2028 2038. (10.10.1002/hep.31599) 33111374

[94] Gaber Y AbdAllah M Salama A Sayed M Abdel Alem S Nafady S . Metabolic-associated fatty liver disease and autoimmune hepatitis: an overlooked interaction. Expert Rev Gastroenterol Hepatol. 2021;15(10):1181 1189. (10.10.1080/17474124.2021.1952867) 34263707

[95] Da BL Ben-Yakov G Kleiner D Koh C . Drug-induced liver injury: understanding the different immune-mediated phenotypes and clinical management. Curr Hepatol Rep. 2018;17(3):235 244. (10.10.1007/s11901-018-0407-9)

[96] deLemos AS Foureau DM Jacobs C Ahrens W Russo MW Bonkovsky HL . Drug-induced liver injury with autoimmune features. Semin Liver Dis. 2014;34(2):194 204. (10.10.1055/s-0034-1375959) 24879983

[97] European Association for the Study of the Liver. EASL Clinical Practice Guidelines: drug-induced liver injury. J Hepatol. 2019;70(6):1222 1261. (10.10.1016/j.jhep.2019.02.014) 30926241

[98] Andrade RJ Aithal GP de Boer YS , et al. Nomenclature, Diagnosis and Management of Drug-induced Autoimmune-like hepatitis (DI-ALH): an expert opinion meeting report. J Hepatol. 2023;79(3):853 866. (10.10.1016/j.jhep.2023.04.033) 37164270 PMC10735171

[99] Chalasani N Li YJ Dellinger A , et al. Clinical features, outcomes, and HLA risk factors associated with nitrofurantoin-induced liver injury. J Hepatol. 2023;78(2):293 300. (10.10.1016/j.jhep.2022.09.010) 36152763 PMC9852026

[100] Suzuki A Brunt EM Kleiner DE , et al. The use of liver biopsy evaluation in discrimination of idiopathic autoimmune hepatitis ­versus drug-induced liver injury. Hepatology. 2011;54(3):931 939. (10.10.1002/hep.24481) 21674554 PMC3192933

[101] Tsutsui A Harada K Tsuneyama K , et al. Histopathological analysis of autoimmune hepatitis with“acute” presentation: differentiation from drug-induced liver injury. Hepatol Res. 2020;50(9):1047 1061. (10.10.1111/hepr.13532) 32515851

[102] Björnsson E Talwalkar J Treeprasertsuk S , et al. Drug-induced autoimmune hepatitis: clinical characteristics and prognosis. Hepatology. 2010;51(6):2040 2048. (10.10.1002/hep.23588) 20512992

[103] Björnsson ES Medina-Caliz I Andrade RJ Lucena MI . Setting up criteria for drug-induced autoimmune-like hepatitis through a systematic analysis of published reports. Hepatol Commun. 2022;6(8):1895 1909. (10.10.1002/hep4.1959) 35596597 PMC9315110

[104] Tujios SR Lee WM . Acute liver failure induced by idiosyncratic reaction to drugs: challenges in diagnosis and therapy. Liver Int. 2018;38(1):6 14. (10.10.1111/liv.13535) 28771932 PMC5741491

[105] Martínez-Casas OY Díaz-Ramírez GS Marín-Zuluaga JI , et al. Differential characteristics in drug-induced autoimmune hepatitis. JGH Open. 2018;2(3):97 104. (10.10.1002/jgh3.12054) 30483571 PMC6207017

[106] Sebode M Schulz L Lohse AW . “Autoimmune(-Like)” drug and herb induced liver injury: new insights into molecular pathogenesis. Int J Mol Sci. 2017;18(9):1954. (10.10.3390/ijms18091954) 28895915 PMC5618603

[107] Westbrook RH Yeoman AD Kriese S Heneghan MA . Outcomes of pregnancy in women with autoimmune hepatitis. J Autoimmun. 2012;38(2-3):J239 J244. (10.10.1016/j.jaut.2011.12.002) 22261501

[108] Rahim MN Ran S Shah S Hughes S Heneghan MA . Safety and efficacy of budesonide during pregnancy in women with autoimmune hepatitis. Hepatology. 2021;73(6):2601 2606. (10.10.1002/hep.31634) 33188708

